# Membrane Proteomics of Arabidopsis Glucosinolate Mutants *cyp79B2/B3* and *myb28/29*

**DOI:** 10.3389/fpls.2017.00534

**Published:** 2017-04-11

**Authors:** Islam Mostafa, Mi-Jeong Yoo, Ning Zhu, Sisi Geng, Craig Dufresne, Maged Abou-Hashem, Maher El-Domiaty, Sixue Chen

**Affiliations:** ^1^Department of Biology, University of FloridaGainesville, FL, USA; ^2^Genetics Institute, University of FloridaGainesville, FL, USA; ^3^Department of Pharmacognosy, Faculty of Pharmacy, Zagazig UniversityZagazig, Egypt; ^4^Plant Molecular and Cellular Biology Program, University of FloridaGainesville, FL, USA; ^5^Thermo Fisher ScientificWest Palm Beach, FL, USA; ^6^Interdisciplinary Center for Biotechnology Research, University of FloridaGainesville, FL, USA

**Keywords:** Arabidopsis, membrane proteome, glucosinolate, stress and defense, molecular networks

## Abstract

Glucosinolates (Gls) constitute a major group of natural metabolites represented by three major classes (aliphatic, indolic and aromatic) of more than 120 chemical structures. In our previous work, soluble proteins and metabolites in Arabidopsis mutants deficient of aliphatic (*myb28/29*) and indolic Gls (*cyp79B2B3*) were analyzed. Here we focus on investigating the changes at the level of membrane proteins in these mutants. Our LC/MS-MS analyses of tandem mass tag (TMT) labeled peptides derived from the *cyp79B2/B3* and *myb28/29* relative to wild type resulted in the identification of 4,673 proteins, from which 2,171 are membrane proteins. Fold changes and statistical analysis showed 64 increased and 74 decreased in *cyp79B2/B3*, while 28 increased and 17 decreased in *myb28/29*. As to the shared protein changes between the mutants, one protein was increased and eight were decreased. Bioinformatics analysis of the changed proteins led to the discovery of three cytochromes in glucosinolate molecular network (GMN): cytochrome P450 86A7 (At1g63710), cytochrome P450 71B26 (At3g26290), and probable cytochrome c (At1g22840). CYP86A7 and CYP71B26 may play a role in hydroxyl-indolic Gls production. In addition, flavone 3′-O-methyltransferase 1 represents an interesting finding as it is likely to participate in the methylation process of the hydroxyl-indolic Gls to form methoxy-indolic Gls. The analysis also revealed additional new nodes in the GMN related to stress and defense activity, transport, photosynthesis, and translation processes. Gene expression and protein levels were found to be correlated in the *cyp79B2/B3*, but not in the *myb28/29*.

## Introduction

Glucosinolates (Gls) as natural anticancer compounds are represented by three major classes of chemical structures (aliphatic, indolic, and aromatic; Yan and Chen, 2007; Sønderby et al., 2010). In addition to their anti-carcinogenic activities, they have a distinct role in plant defense against herbivores (Halkier and Gershenzon, 2006; Yan and Chen, 2007) and pathogens (Kissen et al., 2009). The activities are attributed to their hydrolysis products, such as isothiocyanates, thiocyanates, and nitriles (Halkier and Gershenzon, 2006). Gls biosynthesis starts from methionine, tryptophan or phenylalanine to produce aliphatic, indolic, or aromatic Gls, respectively (Yan and Chen, 2007; Sønderby et al., 2010). Briefly, the substrate amino acid is converted to aldoxime, then to *aci*-nitro compounds, thiohydroximate, and desulfoglucosinolate. After sulfation, the core Gls structure is formed. In aliphatic Gls biosynthesis, the methionine chain-elongation and the core structure biosynthesis are under the control of three transcriptional factors MYB28, MYB29, and MYB76 (Yan and Chen, 2007; Frerigmann et al., 2012). In the core pathway, the formation of aldoximes is catalyzed by cytochrome P450s CYP79F1 and CYP79F2, and that of the *aci*-nitro compounds by CYP83A1 (Grubb and Abel, 2006). Then glutathione S-transferase U20 forms thiohydroximates, which are in turn rearranged to desulfoglucosinolate by UGT74B1 (Sønderby et al., 2010), followed by sulfation by SOT17 and SOT18 to produce intact Gls (Sønderby et al., 2010; Mostafa et al., 2016). Similar for indolic Gls, CYP79B2, CYP79B3, and CYP83B1 are responsible for aldoximes and *aci*-nitro compounds formation, followed by conversion to thiohydroximates by glutathione S-transferase F10, rearrangement to desulfoglucosinolates and sulfation to indolic Gls by SOT16 (Grubb and Abel, 2006; Mostafa et al., 2016). It is clear that the cytochrome P450s play a central role in the Gls biosynthesis, and these proteins are membrane localized (Neve and Ingelman-Sundberg, 2010).

Several studies have reported the relationship between the Gls biosynthetic pathway and other biological pathways in plants, e.g., amino acid and carbohydrate pathways using *CYP79F1* RNAi lines (Chen et al., 2012), auxin biosynthesis using *cyp79B2/B3* mutant (Zhao et al., 2002) and stress response pathways through environmental perturbation (Martínez-Ballesta et al., 2013). In our previous work, we used Arabidopsis double mutants (*cyp79B2/B3* deficient in indolic Gls production and *myb28/29* deficient in aliphatic Gls production), and discovered new nodes in the glucosinolate molecular network (GMN) that include stress and defense related proteins like glucan endo-1,3-beta-glucosidase, glutathione S-transferase F7 and glutathione S-transferase F2 and the electron carriers cytochrome B5 isoform C and cytochrome c oxidase subunit 5b-2 (Mostafa et al., 2016). To date, no studies have reported the glucosinolate molecular networks in the membrane proteome context.

Since many known glucosinolate proteins such as the cytochrome P450s are membrane or membrane associated proteins, here we investigated how perturbation of Gls metabolism using the aforementioned mutants affects the Arabidopsis membrane proteome using Tandem Mass Tag (TMT) labeling LC-MS/MS based quantitative proteomics. Analyses of protein interaction networks using STRING and functional enrichment of the identified proteins using agriGO allowed us to discover new nodes and edges in the GMN. With qRT-PCR, we were able to determine the correlation between gene transcripts and membrane proteins in the two mutants. Together with our published soluble proteomics work (Mostafa et al., 2016), this study enables a comprehensive understanding of the Arabidopsis GMNs.

## Materials and methods

### Plant genotyping, growth, and sample collection

*Arabidopsis thaliana* (L.) Heynh ecotype Columbia (Col-0) seeds were obtained from the Arabidopsis Biological Resource Center (Columbus, OH, USA). The seeds of *cyp79B2/B3* and *myb28/29* were kindly provided by Dr. John Celenza (Boston University, Boston, MA, USA) and Dr. Masami Hirai (RIKEN Plant Science Center, Yokohama, Japan), respectively. The mutant genotyping and chemotyping were reported in our previous study (Mostafa et al., 2016). Seed germination and seedling growth were conducted as previously described (Mostafa et al., 2016). Leaves from 5-week old wild type (WT), *cyp79B2/B3* and *myb28/29* were collected, frozen in liquid nitrogen and stored at −80°C. Four replicates were included per genotype, and each replicate contains 2 g leaves pooled from 12 plants.

### Protein extraction and peptide TMT labeling

Protein was extracted according to Pang et al. (2010) by grinding the leaf tissues in liquid nitrogen and then homogenizing on ice in 10 mM Tris-HCl (pH 7.4), 10 mM KCl, 1.5 mM MgCl_2_, 10 mM dithiothritol (DTT), 0.5 M sucrose, and 10 mM phenylmethylsulfonyl fluoride (PMSF). The protein extracts were filtered through cheesecloth and centrifuged at 800 g for 10 min at 4°C. The supernatant was transferred to ultracentrifuge tubes and centrifuged again at 100,000 g for 1.5 h at 4°C. The formed microsomal membrane was washed with 100 mM sodium carbonate using a glass dounce homogenizer, followed by centrifugation at 100,000 g for 1.5 h at 4°C. The microsome pellets were rinsed with 500 μl resuspension buffer containing 100 mM HEPES (pH 7), 1% triton X-100 and 0.5 M sucrose, and centrifuged at 800 *g* for 10 min at 4°C. Protein was precipitated using 5 volumes ice cold 90% acetone overnight at −20°C, followed by washing the pellets once with ice cold 90% acetone and twice with ice cold acetone before solubilizing in 7 M urea, 2 M thiourea, 4% CHAPS, and 0.25% Triton X-100. The protein amount was assayed using an EZQ assay kit (Invitrogen Inc., Eugene, OR, USA).

A total of 50 μg protein from each replicate was precipitated with ice cold 90% acetone at −20°C overnight, followed by 20,000 *g* centrifugation at 4°C for 15 min. After washing with ice cold 90% acetone, the pellets were solubilized, reduced, alkylated and digested with modified trypsin (Promega, Madison, WI, USA) at a 1:25 (w/w) ratio for 16 h at 37°C, followed by TMT labeling according to the TMT 6-plex kit manual (Thermo Scientific Inc., San Jose, CA, USA). The WT replicates were labeled with 126 and 127 tags, *cyp79B2*/*B3* replicates with 128 and 129 tags and *myb28*/*29* replicates with 130 and 131 tags at room temperature for 2 h. After quenching with 8 μl 5% hydroxylamine for 30 min, the labeled samples were combined and lyophilized. Two independent experiments and four biological replicates each sample were performed.

### Peptide desalting, strong cation exchange fractionation, and LC-MS/MS analysis

The TMT labeled peptides were desalted on Macrospin C-18 reverse phase mini-column (The Nestgroup Inc., Southborough, MA, USA) and fractionated using an Agilent HPLC 1260 strong cation exchange system as previously described (Mostafa et al., 2016). A total of 12 fractions were collected from each experiment. Each fraction was lyophilized, solubilized in solvent A (0.1% formic acid and 3% acetonitrile), and analyzed using an Easy-nLC 1000 system coupled to a Q-Exactive Orbitrap Plus MS (Thermo Fisher Scientific, Bremen, Germany) according to Mostafa et al. (2016) with minor modifications: The mobile phase gradient was ramped from 2 to 30% of solvent B (0.1% formic acid and 99.9% acetonitrile) in 57 min, then to 98% of solvent B in 6 min and maintained for 12 min. Mass analysis was performed in positive ion mode with high collision dissociation energy. The scan range was 400–2,000 *m/z* with full MS resolution of 70,000 and 200–2,000 *m/z* with MS^2^ resolution of 17,500. The first mass was fixed at 115 *m/z*, and 445.12003 *m/z* (polysiloxane ion mass) was used for real-time mass calibration.

### Protein identification and quantification

The MS data were searched using Proteome Discoverer 1.4 (Thermo Scientific, Bremen, Germany) against the *Arabidopsis* TAIR10 database with 35,386 entries. The searching parameters were set to 300 and 5,000 Da as minimum and maximum precursor mass filters, digestion with trypsin with two missed cleavages, Carbamidomethylation of cysteine was set as a static modification, and TMT6plex of N terminus, TMT6plex of lysine, phosphorylation of STY (serine, threonine, and tyrosine) and methionine oxidation were set as dynamic modifications. Precursor mass tolerance was 10 ppm, fragment mass tolerance was 0.01 Da, spectrum grouping maximum retention time difference was 1.1 and false discovery rate was 0.01 at the peptide level. Proteins quantification based on labeled unique peptides intensities and statistical analyses were performed as previously described (Chen et al., 2012; Mostafa et al., 2016; Sun et al., 2017). The proteomics data were deposited to ProteomeXchange repository (accession number: PXD005781).

### String bioinformatics analysis and gene ontology enrichment

The relationship between the significantly changed proteins and Gls metabolic pathways (Chen et al., 2011; Mostafa et al., 2016) was analyzed using STRING bioinformatics tool (Baldrianová et al., 2015; Ji et al., 2016; Lim et al., 2017). The resulted networks were visualized in the confidence view relying on gene neighborhood, fusion, co-occurrence, co-expression, literature, and available data. To determine the enriched pathways, we performed Singular Enrichment Analysis (SEA) for the changed proteins and the results were compared using a cross comparison of SEA (SEACOMPARE) in the agriGO database (Silva-Sanchez et al., 2013).

### Quantitative real-time polymerase chain reaction (qRT-PCR)

To determine whether protein expression levels were correlated with transcript levels, we conducted qRT-PCR of 44 genes selected based on the proteomics data (32 for *cyp79B2/B3* and 22 for *myb28/28*). This list of primers used in qRT-PCR is provided in Supplementary Table 1. Total RNA was extracted using a RNeasy Plant Mini Kit (Qiagen, Valencia, CA, USA) and cDNA was synthesized with ProtoScript® II Reverse Transcriptase (New England BioLabs, Ipswich, MA, USA). qRT-PCR was performed with VeriQuest SyBr and a fluorescein kit (Affymetrix, Santa Clara, CA, USA) using CFX96 (Bio-Rad, Hercules, CA, USA) as described previously (Koh et al., 2012). For each reaction, three technical and three biological replicates were included. Relative expression of the target genes was calculated using the comparative *C*_t_ method (Applied Biosystems, Framingham, USA). The differences in *C*_t_ values (Δ*C*_t_) between the target gene and two internal controls (*AT4G34270* and *AT5G44200*) were calculated to normalize differences in the starting materials. The expression ratios of *cyp79B2/B3* and *myb28/29* to WT were calculated and compared to the ratios from the protein data using Pearson's *r*.

## Results

### *cyp79B2/B3* and *myb28/29* membrane proteomes

**Figure 1:**
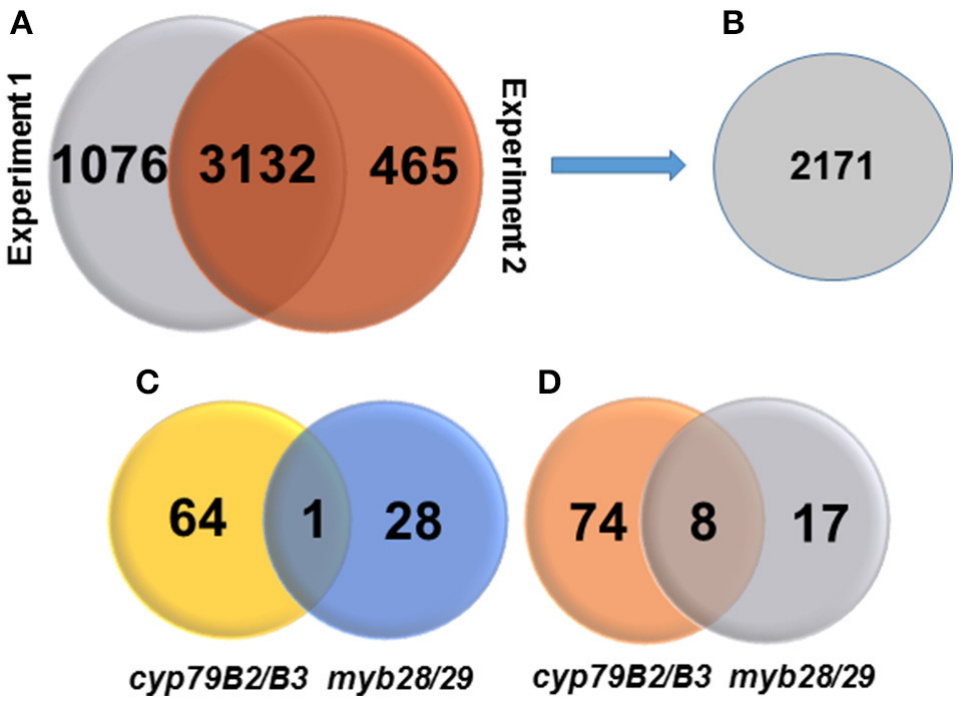
**Venn diagrams showing numbers of identified proteins, membrane proteins, changed proteins, and their distributions. (A)** Number of identified proteins in two independent TMT experiments at high peptide confidence. **(B)** Number of identified membrane proteins. **(C)** Number of significantly increased membrane proteins in *cyp79B2/B3* and *myb28/29* relative to WT at *p* < 0.05 and fold change >1.2. **(D)** Number of significantly decreased membrane proteins in *cyp79B2/B3* and *myb28/29* relative to WT at *p* < 0.05 and fold change < 0.8.

**Figure 2:**
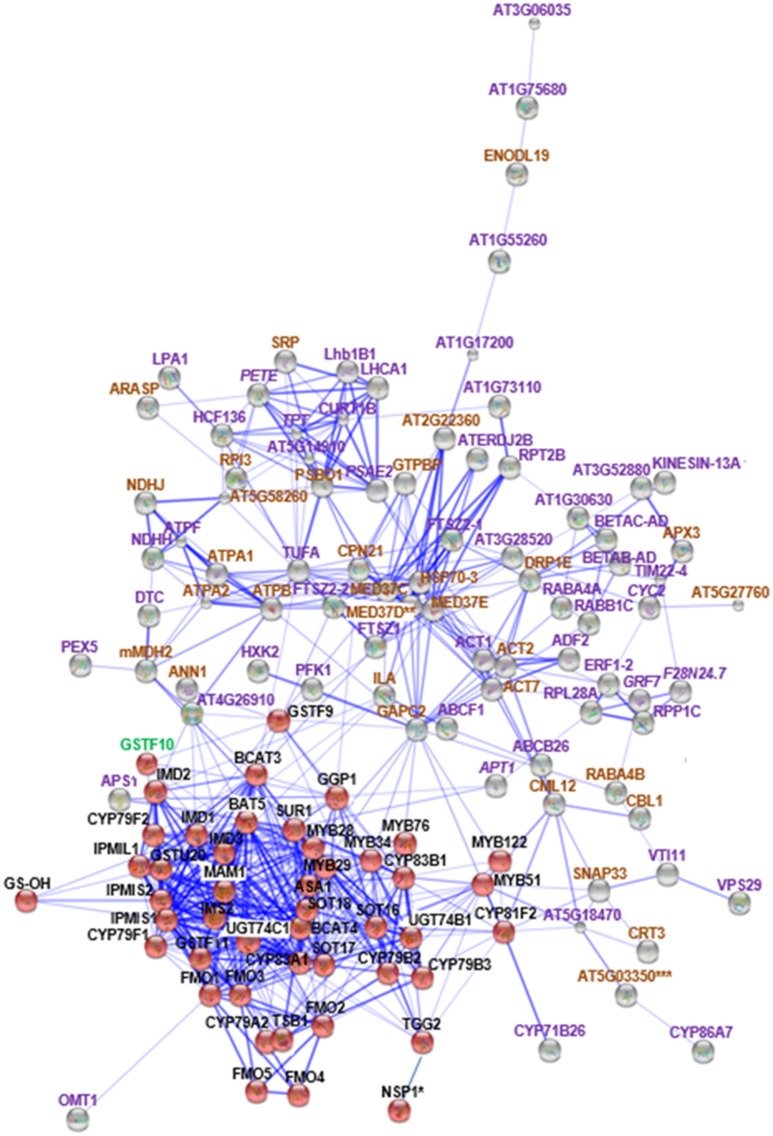
**STRING analysis of ***cyp79B2/B3*** changed proteins in relation to known proteins in Gls biosynthesis**. Known Gls biosynthetic proteins are indicated by red balls, new proteins in the GMN are indicated by gray balls, proteins changed in both mutants are indicated by italic labeling, and uniquely changed proteins in *cyp79B2/B3* are indicated by non-italic labeling. Proteins involved in Gls biosynthesis, stress and defense, and other processes are labeled with green, brown, and violet labels, respectively. Connections strength are proportional to edges thickness as derived from neighborhood, gene fusion, co-occurrence, co-expression, previous experiments, and text-mining information at medium confidence score. Asterisk (^*^) indicates manual connections based on literature. Double asterisk (^**^) indicates known nodes in both mutants (Mostafa et al., 2016), and triple asterisk (^***^) indicates known nodes in *cyp79B2/B3* (Mostafa et al., 2016). Full names of the mapped proteins can be found in the abbreviation and protein name columns in Tables 1, 2.

**Figure 3:**
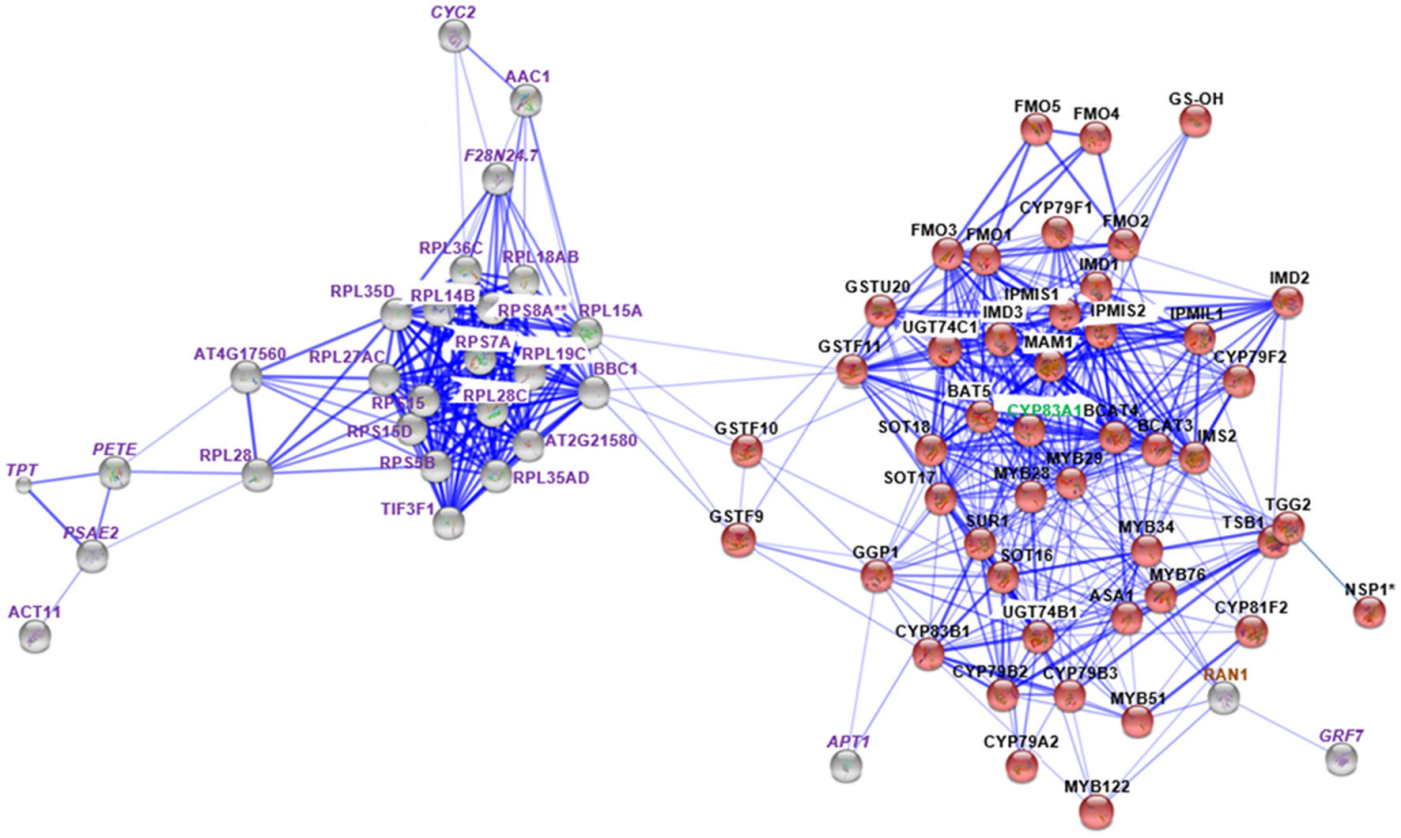
**STRING analysis of ***myb28/29*** changed proteins in relation to known proteins in Gls biosynthesis**. Known Gls biosynthetic proteins are indicated by red balls, new proteins in GMN are indicated by gray balls, proteins changed in both mutants are indicated by italic labeling, and uniquely changed proteins in *myb28/29* are indicated by non-italic labeling. Proteins involved in Gls biosynthesis, stress and defense, and other processes are labeled with green, brown, and violet labels, respectively. Connections strength are proportional to edges thickness as derived from neighborhood, gene fusion, co-occurrence, co-expression, previous experiments, and text-mining information at medium confidence score. Asterisk (^*^) indicates manual connections based on literature. Double asterisk (^**^) indicates known nodes in both mutants (Mostafa et al., 2016). Full names of the mapped proteins can be found in the abbreviation and protein name columns in Tables 1, 2.

Based on the MS/MS spectra of high confidence peptides derived from the WT, *cyp79B2/B3* and *myb28/29*, we identified 4673 proteins in two independent experiments using Proteome Discover (Supplementary Table 2). Out of these proteins, 3,132 were identified in both experiments, while 1,076 and 465 were unique to experiments 1 and 2, respectively (Figure 1A). A total of 4,655 proteins were available for quantification based on unique TMT labeled peptides, highlighting the high efficiency of labeling. PD enrichment analysis (based on TAIR and Uniprot annotations) of the identified proteins showed 2,171 to be membrane proteins (Figure 1B and Supplementary Table 2). Comparative analysis of the protein expression changes between the mutants and WT at a fold change cutoff (>1.2 and <0.8), a *p* < 0.05 and transmembrane domain analysis revealed 93 proteins to be increased (Figure 1C) and 99 to be decreased (Figure 1D). Transmembrane domain analysis revealed that 175 out of the 192 differentially expressed proteins contained at least one transmembrane domains. The rest deemed to be membrane associated proteins (Supplementary Table 3). Correlating the changed proteins to those involved in Gls metabolism using STRING showed new nodes and edges (Figures 2, 3). The new nodes can be categorized according to their positions in the network as directly correlated or indirectly correlated to Gls metabolism. They can also be classified according to their biological roles as secondary (stress related) and tertiary (other biological process) connections (Detailed in next sections).

### Common changes of membrane proteins between the *cyp79B2/B3* and *myb28/29*

Nine membrane proteins showed common changes between the two mutants relative to WT, with only one protein increased while the other eight decreased (Table 1). By STRING mapping of the significantly changed proteins (Figures 2, 3), we found seven of the nine proteins represented new connections with the glucosinolate metabolic network (GMN). The role of probable cytochrome c (CYC2) and plastocyanin minor isoform (PETE) in electron transport process (Pesaresi et al., 2009; Welchen et al., 2012) makes them biologically relevant tertiary connections in GMN in a way similar to cytochrome B5 isoform C and cytochrome c oxidase subunit 5b-2 (Mostafa et al., 2016). Photosystem I reaction center subunit IV B (PSAE2), 14-3-3-like protein GF14 nu (GRF7), adenine phosphoribosyltransferase 1 (APT1), alba DNA/RNA-binding protein (F28N24.7) and triose phosphate/phosphate translocator (APE2) form other tertiary nodes. Out of this group, APT1 was the only protein directly connected to the GMN (Figures 2, 3).

**Table 1:** **List of common membrane proteins showing significant changes in ***cyp79B2/B3*** and ***myb28/29*** mutants relative to WT and their biological functions**.

**Accession number**	**Locus**	**Protein name**	Abbreviation^*^	**FC *cyp*^a^**	***p*-value *cyp*^b^**	**FC *myb*^a^**	***p*-value *myb*^b^**	**Function**	**TMDs tool**	**References**
Q9S714	At2g20260	Photosystem I reaction center subunit IV B	PSAE2	1.640	0.016	1.359	0.044	Photosynthesis	D, H, T	Tair
F4I6B4	At1g30470	SIT4 phosphatase-associated family protein	AT1G30470	0.782	0.030	0.781	0.049	Phosphatase reactions	D, H, T	Tair
Q96300	At3g02520	14-3-3-like protein GF14 nu	GRF7	0.755	0.006	0.677	0.002	Binding of protein with phosphor ylated amino acids	D, T	Tair
Q9LJX0	At3g28860	ABC transporter B family member 19	ABCB19	0.720	0.019	0.753	0.009	Auxin transport	D, H, S, T, M	Lin and Wang, 2005
P31166	At1g27450	Adenine phosphoribosyltransferase 1	APT1	0.716	0.016	0.689	0.043	Adenine phosphorylation	D, H, T	Allen et al., 2002
Q9LP53	At1g29250	Alba DNA/RNA-binding protein	F28N24.7	0.680	0.027	0.527	0.008	Binding of nucleic acid	D, H, S, T	Tair
F4KG20	At5g46110	Triose phosphate/phosphate translocator TPT	APE2	0.608	0.005	0.784	0.043	Transport of triose phosphate	D, H, S, T, M	Tair
O23138	At1g22840	Probable cytochrome c	CYC2	0.356	0.000	0.517	0.037	Electron transport	Integral/peripheral membrane protein	Birchmeier et al., 1976; Welchen et al., 2012
P11490	At1g76100	Plastocyanin minor isoform	PETE	0.186	0.031	0.185	0.028	Electron transport	D, H, S, T	Pesaresi et al., 2009

* *Abbreviations for the shared proteins in Figures 2, 3*.

a *Fold change at cut-off point >1.2 or < 0.8*.

b *p < 0.05*.

*TMDs, transmembrane domains; D, Das; H, HMMTOP; S, SOSUI; T, TMPred; M, TMHMM*.

### Specific changes of *cyp79B2/B3* membrane proteins

Sixty-four and 74 membrane proteins showed unique increases and decreases, respectively, in the *cyp79B2/B3* mutant (Table 2). Seventy-seven new nodes were discovered by the STRING mapping of these *cyp79B2/B3* proteins to the GMN (Figure 2). It was obvious that perturbation of the indolic Gls metabolism affected a group of stress-related membrane proteins forming new secondary nodes. Representative examples from this group are calmodulin-like protein 12 (CML12; Cazzonelli et al., 2014), mediator of RNA polymerase II transcription subunit 37c (MED37C; Lee et al., 2009), SNAP25 homologous protein (SNAP33; Eschen-Lippold et al., 2012), dynamin-related protein 1E (DRP1E; Minami et al., 2015), protein ILITYHIA (ILA; Monaghan and Li, 2010), glyceraldehyde-3-phosphate dehydrogenase (GAPC2; Guo et al., 2012), L-ascorbate peroxidase 3 (APX3; Narendra et al., 2006), Ras-related protein (RABA4B; Antignani et al., 2015), annexin D1 (ANN1; Gorecka et al., 2005; Jia et al., 2015), hypoxia-responsive family protein (At5g27760), and malate dehydrogenase 2 (mMDH2; Jones et al., 2006).

**Table 2:** **List of membrane proteins in the ***cyp79B2/B3*** mutant showing significant changes relative to WT and their biological functions**.

**Accession**	**Locus tag**	**Protein name**	Abbreviation^*^	**FC^a^**	***p*-value^b^**	**Function**	**TMDs**	**References**
Q42545	At5g55280	Cell division protein FtsZ homolog 1	FTSZ1	2.225	0.016	Division of chloroplast and protein binding	D, T	Osteryoung et al., 1998
P25071	At2g41100	Calmodulin-like protein 12	CML12	2.074	0.012	Stimuli response	D, T	Cazzonelli et al., 2014
Q9S726	At3g04790	Probable ribose-5-phosphate isomerase 3	RPI3	2.034	0.005	Bacterial response and management of pentose phosphate cycle	D, H, T	Jones et al., 2006
O82533	At2g36250	Cell division FtsZ homolog 2-1	FTSZ2-1	2.032	0.012	Division of chloroplast and protein binding	D, H, T	Osteryoung et al., 1998
Q9LXJ0	At3g52750	Cell division FtsZ homolog 2-2	FTSZ2-2	1.823	0.031	Division of chloroplast and protein binding	D, H, T	McAndrew et al., 2008
O82660	At5g23120	Photosystem II stability/assembly factor HCF136	HCF136	1.770	0.010	Photosynthesis process	D, H, T	Meurer et al., 1998
Q94K76	At5g18470	Curculin-like (Mannose-binding) lectin family protein	AT5G18470	1.769	0.035	Binding of carbohydrate	D, H, S, T, M	Tair
Q93VK7	At5g14910	At5g14910/F2G14_30	AT5G14910	1.752	0.036	Metal transport	Memebrane associated	Tair, Friso et al., 2004
P53492	At5g09810	Actin-7	ACT7	1.752	0.004	Stress response and growth	D, T	Jelenska et al., 2014
P56757	AtCg00120	ATP synthase subunit alpha	ATPA1	1.750	0.044	Bacterial and cold response and synthesis of ATP	T	Jones et al., 2006
F4JRH9	At4g12880	Early nodulin-like protein 19	ENODL19	1.715	0.010	Stimuli response and electron carrier	D, H, S, T, M	Tair
P22954	At5g02490	Probable mediator of RNA polymerase II subunit 37c	MED37D	1.683	0.017	Bacterial, viral and heat response and transcription control	D, H, T	Uniprot
P0CJ46	At2g37620	Actin-1	ACT1	1.676	0.003	Growth and ATP binding	D, T	Kandasamy et al., 2002; tair
P19366	AtCg00480	ATP synthase subunit beta	ATPB	1.661	0.050	Fungal and cold response and ATP metabolism	D, T	Mukherjee et al., 2010; tair
Q9AST9	At1g73110	At1g73110/F3N23_39	AT1G73110	1.614	0.036	Hydrolysis process and binding of ATP	D, H, T	Tair
Q8LEQ0	At5g47700	60S acidic ribosomal protein P1-3	RPP1C	1.594	0.023	Translation and binding of protein	D, H, T	Tair
Q9SUS3	At4g11380	Beta-adaptin-like protein B	BETAB-AD	1.588	0.010	Transport of protein	D, T	Tair
F4HR88	At1g33590	Leucine-rich repeat (LRR) protein	AT1G33590	1.582	0.004	Defense process	D, H, T, M	Ascencio-Ibáñez et al., 2008
Q9LPV8	At1g12920	Eukaryotic peptide chain release factor subunit 1-2	ERF1-2	1.577	0.027	Termination of translation	D, T	Tair
Q8H107-3	At4g26910	Isoform 3 of dihydrolipoyllysine-residue succinyltransferase	AT4G26910	1.577	0.024	L-lysine catabolism and a member of tricarboxylic acid cycle	D, H, T	Tair
P17745	At4g20360	Elongation factor Tu	TUFA	1.563	0.006	Translation, binding of GTP and Cys nitrosylation	D, T	Tair
Q9LHA8	At3g12580	Mediator of RNA polymerase II transcription subunit 37c	MED37C	1.543	0.003	Response to stress	D, T	Lee et al., 2009
Q940B8	At3g16630	Kinesin-13A	KINESIN-13A	1.524	0.027	Binding of ATP	D, H	Tair
F4HW29	At1g08450	Calreticulin-3	CRT3	1.520	0.010	Defense process	D, H, S, T, M	Sun et al., 2014
P23321	At5g66570	Oxygen-evolving enhancer protein	PSBO1	1.514	0.019	Bacterial response and photosynthesis process	D, H, T	Tair; Murakami et al., 2002
Q8L940	At5g01410	Pyridoxal biosynthesis protein PDX1.3	PDX13	1.467	0.020	Stress response	T	Czégény et al., 2014
Q96292	At3g18780	Actin-2	ACT2	1.463	0.003	Red light response and root growth	D, T	Kandasamy et al., 2002; tair
Q8VZC7-2	At5g45510	Isoform 2 of Probable disease resistance protein	AT5G45510	1.455	0.018	Defense process	D, T	Desveaux et al., 2005
Q9LET7	At3g56690	Calmodulin-interacting protein 111	CIP111	1.442	0.042	Hydrolysis and binding of ATP	D, T	Tair
Q8LCA1	At2g46820	Protein curvature thylakoid 1B	CURT1B	1.436	0.035	Photosynthesis and DNA binding	D, H, S, T, M	Tair
Q9LZF5	At5g03350	Lectin-like protein At5g03350	AT5G03350	1.426	0.010	Salicylic acid and immunological response	D, H, T	Armijo et al., 2013
O81742	At4g23460	Beta-adaptin-like protein C	BETAC-AD	1.417	0.016	Transport of protein	D, T	Tair
Q9FJH6	At5g60790	ABC transporter F family member 1	ABCF1	1.404	0.002	Transport process and Binding of ATP	D, H, T	Tair
Q39251	At3g46000	Actin-depolymerizing factor 2	ADF2	1.399	0.001	Depolymerization of actin	Membrane	Tair and Abe et al., 1996
O22265	At2g47450	Signal recognition particle 43 kDa	CAO	1.396	0.020	Response to light	D, T	Walter et al., 2015
P56753	AtCg01110	NAD(P)H-quinone oxidoreductase subunit H	NDHH	1.374	0.034	Photosynthesis and oxidation reduction activities	D, T	Tair
Q9SRY4	At1g02910	Protein low PSII accumulation 1	LPA1	1.368	0.016	Member of photosystem	D, H, S, T, M	Peng et al., 2006
Q9SL67	At2g20140	26S proteasome regulatory subunit 4 homolog B	RPT2B	1.361	0.007	Hydrolysis of ATP and generation of gametes	D, T	Tair
Q9FXA1	At1g49750	At1g49750 protein	AT1G49750	1.340	0.034		D, H, S, T	Tair
A8MS75	At3g54890	Light-harvesting complex I chlorophyll a/b binding protein 1	LHCA1	1.339	0.018	Photosynthetic process	D, H, S, T	Tair
Q9SR77	At3g10130	Heme-binding-like protein	AT3G10130	1.330	0.024	Binding of heme	D, T	Tair
P22953	At5g02500	Probable mediator of RNA polymerase II subunit 37e	MED37E	1.328	0.004	Immunity response	D, T	Noël et al., 2007
F4ISI7	At2g19480	Nucleosome assembly protein 12	NAP1; 2	1.318	0.027	Repair and binding of DNA	D	Iglesias et al., 2013
Q9LIK9	At3g22890	ATP sulfurylase 1	APS1	1.305	0.039	Biosynthesis of hydrogen sulfide	D, T	Tair
Q9SJZ7	At2g22360	Molecular chaperone DnaJ	AT2G22360	1.304	0.025	Heat response and binding activity	D, T	Tair
O65719	At3g09440	Heat shock 70 kDa protein 3	HSP70-3	1.300	0.030	Viral and heat response and binding activity	D, T	Agudelo-Romero et al., 2008; Palmblad et al., 2008; Tair
B3H5R4	At5g58260	At5g58260 protein	AT5G58260	1.298	0.009	Fungal response and oxidation reduction reactions	D	Mukherjee et al., 2010; Tair
Q9FMA3	At5g56290	Peroxisome biogenesis protein 5	PEX5	1.294	0.017	Movement of proteins to peroxisome	D, T	Ramón and Bartel, 2010
O80885	At2g32480	Arabidopsis serin protease	ARASP	1.274	0.038	Proteolytic action and stress response	D, H, S, T, M	Sokolenko et al., 2002
Q42044	At2g45180	Bifunctional inhibitor/lipid-transfer protein/seed storage 2S albumin	AT2G45180	1.271	0.032	Proteolytic action and lipid transport	D, H, S, T, M	Tair
F4JTP5	At4g38470	ACT-like protein tyrosine kinase	AT4G38470	1.270	0.042	Kinase and binding activities	D, H, T	Tair
Q9S7P9	At5g61210	SNAP25 homologous protein	SNAP33	1.264	0.035	Immunity process	D	Eschen-Lippold et al., 2012
Q9M0F9	At4g29220	6-phosphofructokinase 1	PFK1	1.262	0.043	Fructose 6 phosphate metabolism and glycolysis	D, T	Mustroph et al., 2007
Q8L7L0	At5g18570	GTP-binding protein OBGC	OBGL	1.250	0.010	Stimuli response	D, T	Chen et al., 2014
Q9FNX5	At3g60190	Dynamin-related protein 1E	DRP1E	1.246	0.005	Cold response	D, T	Minami et al., 2015; tair
Q8RY46	At1g70610	ABC B family member 26	ABCB26	1.237	0.007	Transport activity	D, H, S, T, M	Tair
Q39142	At2g34430	Light-harvesting chlorophyll protein complex II subunit B1	Lhb1B1	1.232	0.049	Photosynthetic process	D, H, S, T	Tair
P92549	AtMg01190	ATP synthase subunit alpha	ATPA2	1.227	0.005	Oxidative stress response	D, T	Sweetlove et al., 2002
Q9SA78	At1g30630	Coatomer subunit epsilon-1	AT1G30630	1.226	0.006	Transport process	D, T	Tair
F4J0B1	At3g28520	AAA-type ATPase family protein	AT3G28520	1.226	0.039	Binding and hydrolysis of ATP	D, T	Tair
Q9ZPH9	At4g00750	Probable methyltransferase PMT15	AT4G00750	1.218	0.024	Stress response and methylation process	D, H, S, T, M	Rama Devi et al., 2006; tair
F4I894	At1g64790	Protein ILITYHIA	ILA	1.217	0.017	Immunity process	D, H, S, T	Monaghan and Li, 2010
O49636	At4g22310	At4g22310	AT4G22310	1.213	0.007	Transport of pyruvate	D, T	Tair
P56754	Atcg00420	NAD(P)H-quinone oxidoreductase subunit J	NDHJ	1.207	0.036	Oxidation reduction and response to sulfur deficiency	D, T	Tair
Q9FX54	At1g13440	Glyceraldehyde-3-phosphate dehydrogenase GAPC2	GAPC2	0.798	0.013	Stress response	D, T	Guo et al., 2012
P19456	At4g30190	ATPase 2, plasma membrane-type	AHA2	0.798	0.048	ATP metabolism	D, H, S, T, M	Tair
P42761	At2g30870	Glutathione S-transferase F10	GSTF10	0.798	0.025	Indolic glucosinolate biosynthesis	D, H, T	Mostafa et al., 2016
Q9LNH6	At1g48240	Novel plant SNARE 12	NPSN12	0.793	0.006	Transport of protein	D, H, S, T, M	Tair
F4ICF5	At1g25290	RHOMBOID-like protein 10	RBL10	0.790	0.001	Root and flower growth	D, H, T, M	Thompson et al., 2012
O65282	At5g20720	20kDa chaperonin	CPN21	0.789	0.021	Defense process	Plasma membrane	Kawamura and Uemura, 2003, Takáč et al., 2014
Q84MC0	At3g06035	Uncharacterized GPI-anchored protein	AT3G06035	0.788	0.035	Precursor for glycoprotein	D, H, S, T, M	Tair
Q9FK25	At5g54160	Flavone 3'-O-methyltransferase 1	OMT1	0.787	0.003	Flavonoid metabolism	D, T	Muzac et al., 2000
Q9FJN8	At5g65270	Ras-related protein RABA4a	RABA4A	0.786	0.003	Binding of GTP and pollen tube growth	D, T	Tair; Szumlanski and Nielsen, 2009
P92963	At4g17170	Ras-related protein RABB1c	RABB1C	0.785	0.029	Binding of GTP and transport activity	D, T	Tair
P56759	Atcg00130	ATP synthase subunit b	ATPF	0.783	0.012	Respiration process	D, H, T, M	Tair
P93834	At2g19860	Hexokinase-2	HXK2	0.782	0.047	Phosphorylation of hexoses	D, H, S, T, M	Jang et al., 1997
Q9CAD6	At1g63710	Cytochrome P450 86A7	CYP86A7	0.781	0.023	Oxidation reduction and metabolism of fatty acid	D, H, S, T, M	Duan and Schuler, 2005
O81016	At2g26910	ABC transporter G family member 32	ABCG32	0.780	0.037	Transport activity and cuticle formation	D, H, S, T, M	Tair; Bessire et al., 2011
A8MQG9	At1g73650	Uncharacterized protein	AT1G73650	0.779	0.021	Oxidation reduction reactions and lipid metabolism	D, H, S, T, M	Tair
Q9C6X2	At1g32050	Secretory carrier-associated membrane protein 4	SCAMP4	0.778	0.025	Carrier activity	D, H, S, T, M	Law et al., 2012
Q96282	At5g49890	Chloride channel protein CLC-c	CLC-C	0.777	0.002	Salt stress	D, H, S, T, M	Jossier et al., 2010
A8MQG9	At1g73650	Uncharacterized protein	AT1G73650	0.779	0.021	Oxidation reduction reactions and lipid metabolism	D, H, S, T, M	Tair
Q9C6X2	At1g32050	Secretory carrier-associated membrane protein 4	SCAMP4	0.778	0.025	Carrier activity	D, H, S, T, M	Law et al., 2012
Q96282	At5g49890	Chloride channel protein CLC-c	CLC-C	0.777	0.002	Salt stress	D, H, S, T, M	Jossier et al., 2010
Q9STT2	At3g47810	Vacuolar protein sorting-associated protein 29	VPS29	0.776	0.044	Transport activity	Membrane associated	Jaillais et al., 2007; Zelazny et al., 2013
Q42564	At4g35000	L-ascorbate peroxidase 3	APX3	0.775	0.041	Antioxidant action and stress response	D, H, T, M	Narendra et al., 2006; tair
Q8VZM7	At5g02940	Putative ion channel POLLUX-like 1	AT5G02940	0.774	0.043	Transport of ions	D, H, S, T, M	Tair
Q8LB17	At3g58460	Uncharacterized protein	AT3G58460	0.774	0.030	Proteolytic action	D, H, S, T, M	Knopf and Adam, 2012
Q8LCP6	At1g75680	Endoglucanase 10	AT1G75680	0.773	0.000	Cellulase and hydrolase action	D, H, S, T, M	Tair
Q94CI7	At5g27350	Sugar transporter ERD6-like 17	SFP1	0.772	0.012	Carbohydrate transport	D, H, S, T, M	Quirino et al., 2001
Q39099	At2g06850	Xyloglucan endotransglucosylase/ hydrolase protein 4	XTH4	0.770	0.027	Stimuli response, cell wall development and hydrolase action	D, H, S, T, M	Campbell and Braam, 1999; tair
Q944A7	At4g35230	Probable serine/threonine-protein kinase	AT4G35230	0.763	0.026	Immunity process and phosphorylation of proteins	D, T	Shi et al., 2013; tair
Q949R9	At5g20090	Mitochondrial pyruvate carrier 1	AT5G20090	0.763	0.046	Transport of pyruvate	D, H, T	Li et al., 2014
Q9LTL0	At3g26290	Cytochrome P450 71B26	CYP71B26	0.760	0.028	Binding of oxygen	D, H, S, T, M	Tair
Q9SMQ6	At4g39990	Ras-related protein RABA4b	RABA4B	0.754	0.017	Defense process	D, T	Antignani et al., 2015
Q9C5M0	At5g19760	Dicarboxylate/tricarboxylate transporter DTC	DTC	0.750	0.021	Dicarboxylate transport	D, H, T	Picault et al., 2002
O82204	At2g19730	60S ribosomal protein L28-1	RPL28A	0.748	0.032	Translation		Tair
Q9LFA3	At3g52880	Probable monodehydroascorbate reductase isoform 3	AT3G52880	0.745	0.024	Oxidation reduction	D, H, T	Lisenbee et al., 2005
Q9SEL6	At5g39510	Vesicle transport v-SNARE 11	VTI11	0.740	0.001	Transport	D, H, S, T, M	Tair
Q9LPZ3	At1g11410	G-type lectin S-receptor-like serine/threonine-protein kinase	AT1G11410	0.733	0.049	Kinase and binding activities	D, H, S, T, M	Tair
Q8LE26	At2g38480	CASP-like protein At2g38480	AT2G38480	0.729	0.002		D, H, T, M	Tair
P43287	At2g37170	Aquaporin PIP2-2	PIP2-2	0.725	0.011	Water deficiency response and transport	D, H, S, T, M	Javot, 2003; Tournaire-Roux et al., 2003;
Q9SYT0	At1g35720	Annexin D1	ANN1	0.719	0.046	Salt stress, binding and transport activities	D	Gorecka et al., 2005; Jia et al., 2015
Q9M1E7	At3g45600	Tetraspanin-3	TET3	0.719	0.042	Member of aging process	D, H, S, T, M	Tair
Q39101	At5g01600	Ferritin-1	FER1	0.713	0.027	Bacterial and stress response and iron homeostasis	D, H, T	Tair
F4JP88	At4g17615	Calcineurin B-like protein 1	CBL1	0.712	0.029	Stress response	Associated with membrane kinase	Ren et al., 2013; Feng et al., 2015
F4JIN3	At4g21180	DnaJ / Sec63 Brl domains-containing protein	ATERDJ2B	0.712	0.044	Transport of protein	D, H, S, T, M	Tair
O23482	At4g16370	Oligopeptide transporter 3	OPT3	0.705	0.035	Transport activity	D, H, S, T, M	Wintz et al., 2003
Q9C8G5	At1g30360	Early-responsive to dehydration stress protein	T4K22.4	0.703	0.007	Water deficiency (stress) response and ion transport	D, H, S, T, M	Rai et al., 2016; tair
Q8L8Z1	At4g15630	CASP-like protein At4g15630	AT4G15630	0.701	0.024	Binding of protein	D, H, S, T, M	Tair
Q9FYK0	At1g24650	Leucine-rich repeat protein kinase F21J9.31	LRR-RLK	0.701	0.043	Growth process.	D, S, T, M	Dai et al., 2013
Q8GWP3	At2g26975	Copper transporter 6	COPT6	0.700	0.005	Transport of copper	D, H, S, T, M	Garcia-Molina et al., 2013
Q8LG60	At5g27760	Hypoxia-responsive family protein	AT5G27760	0.698	0.029	Oxygen deficiency response	D, H, T	Tair
Q9FF88	At5g23920	At5g23920	AT5G23920	0.695	0.038	……	D, H, S, T, M	Tair
Q8LAA6	At4g23400	Probable aquaporin PIP1-5	PIP1-5	0.694	0.049	Controls water channels, salt stress response	D, H, S, T, M	Weig et al., 1997; Tair
Q93XY5	At2g20230	Tetraspanin-18	TOM2AH2	0.690	0.049	….…	D, H, S, T, M	Tair
P30302	At2g37180	Aquaporin PIP2-3	PIP2-3	0.685	0.003	Salt stress and water deficiency	D, H, S, T, M	Daniels et al., 1994
Q8RWZ6	At2g01420	Auxin efflux carrier component 4	PIN4	0.683	0.019	Transport of auxin	D, H, S, T, M	Zhang et al., 2015
A1XJK0	At1g18320	Mitochondrial inner membrane translocase subunit TIM22-4	TIM22-4	0.668	0.044	Transport of protein	D, H, S, T, M	Tair
Q39196	At4g00430	Probable aquaporin PIP1-4	PIP1-4	0.667	0.006	Water deficiency response and transport	D, H, S, T, M	Li et al., 2015; Tair
Q9LZI2	At3g62830	UDP-glucuronic acid decarboxylase 2	UXS2	0.664	0.049	Xylose metabolism	D, H, S, T, M	Harper and Bar-Peled, 2002
Q9LIL4	At3g22845	Transmembrane emp24 domain-containing protein p24beta3	AT3G22845	0.652	0.038	Transport activity	D, H, T, M	Tair
Q9CAN1	At1g63120	RHOMBOID-like protein 2	F16M19.4	0.646	0.018	Proteolytic activity	D, H, S, T, M	Kanaoka et al., 2005
Q9SUV2	At4g32390	Probable sugar phosphate/phosphate translocator	AT4G32390	0.640	0.019	Transport activity	D, H, S, T, M	Tair
Q8GYN5	At3g25070	RPM1-interacting protein 4	RIN4	0.633	0.043	Bacterial response and immunity process	D	Axtell and Staskawicz, 2003
Q9LVE0	At3g21670	Protein NRT1/ PTR FAMILY 6.4	NPF6.4	0.628	0.031	Transport activity and nitrate synthesis	D, H, S, T, M	Okamoto et al., 2003
Q9LFS3	At5g16010	3-oxo-5-alpha-steroid 4-dehydrogenase family protein	F1N13_150	0.627	0.007	Oxidation reduction reactions and lipid metabolism	D, H, S, T, M	Tair
Q9FQ24	At3g55005	Protein TONNEAU 1b	TON1B	0.617	0.001	Growth process and organization of microtubule	D, T	Azimzadeh et al., 2008
B9DFR9	At2g45960	Plasma membrane intrinsic protein 1B, At2g45960 protein	PIP1B	0.615	0.005	Water deficiency	D, H, S, T, M	Alexandersson et al., 2005
O23596	At4g17550	Putative glycerol-3-phosphate transporter 4	AT4G17550	0.613	0.009	Transport activity	D, H, S, T, M	Tair
Q9LVM5	At5g58220	Allantoin synthase/Uric acid degradation bifunctional protein	TTL	0.611	0.030	Cell growth control, allantoin biosynthesis and catabolism of urate	D, H, T	Tair
Q08733	At1g01620	Aquaporin PIP1-3	PIP1-3	0.601	0.020	Water deficiency response and transport	D, H, S, T, M	Kammerloher et al., 1994
Q8VZQ3	At1g17200	CASP-like protein At1g17200	AT1G17200	0.590	0.016	Binding activity	D, H, S, T, M	Tair
Q9M386	At3g54200	Late embryogenesis abundant hydroxyproline-rich glycoprotein	F24B22.160	0.588	0.004	….…	D, H, S, T, M	Tair
F4I082	At1g55260	Glycosylphosphatidylinositol-anchored lipid protein transfer 6	AT1G55260	0.583	0.002	Binding and transport of lipid	D, T	Edstam and Edqvist, 2014
Q9FN38	At5g53880	Putative uncharacterized protein	AT5G53880	0.576	0.012	……	D, T	Tair
F4JDN8	At3g26700	Protein kinase family protein	AT3G26700	0.544	0.025	Kinase action	D, H, S, T, M	Tair
Q9ZV07	At2g39010	Probable aquaporin PIP2-6	PIP2-6	0.531	0.022	Controls water channels	D, H, S, T, M	Alexandersson et al., 2010
P93004	At4g35100	Aquaporin PIP2-7	PIP2-7	0.526	0.032	Salt stress response	D, H, S, T, M	Weig et al., 1997
F4JY28	At5g18630	Putative class 3 lipase	AT5G18630	0.513	0.008	Lipid metabolism	D, H, S, T, M	Tair
A8MQK3	At3g15020	Malate dehydrogenase 2	mMDH2	0.414	0.028	Bacterial response and carbohydrate metabolism	D, H, T	Jones et al., 2006, Tair

* *Abbreviations for unique proteins in Figure 2*.

a *Fold change at cut-off point >1.2 or < 0.8*.

b *p < 0.05*.

*TMDs, transmembrane domains; D, Das; H, HMMTOP; S, SOSUI; T, TMPred; M, TMHMM*.

**Figure 4:**
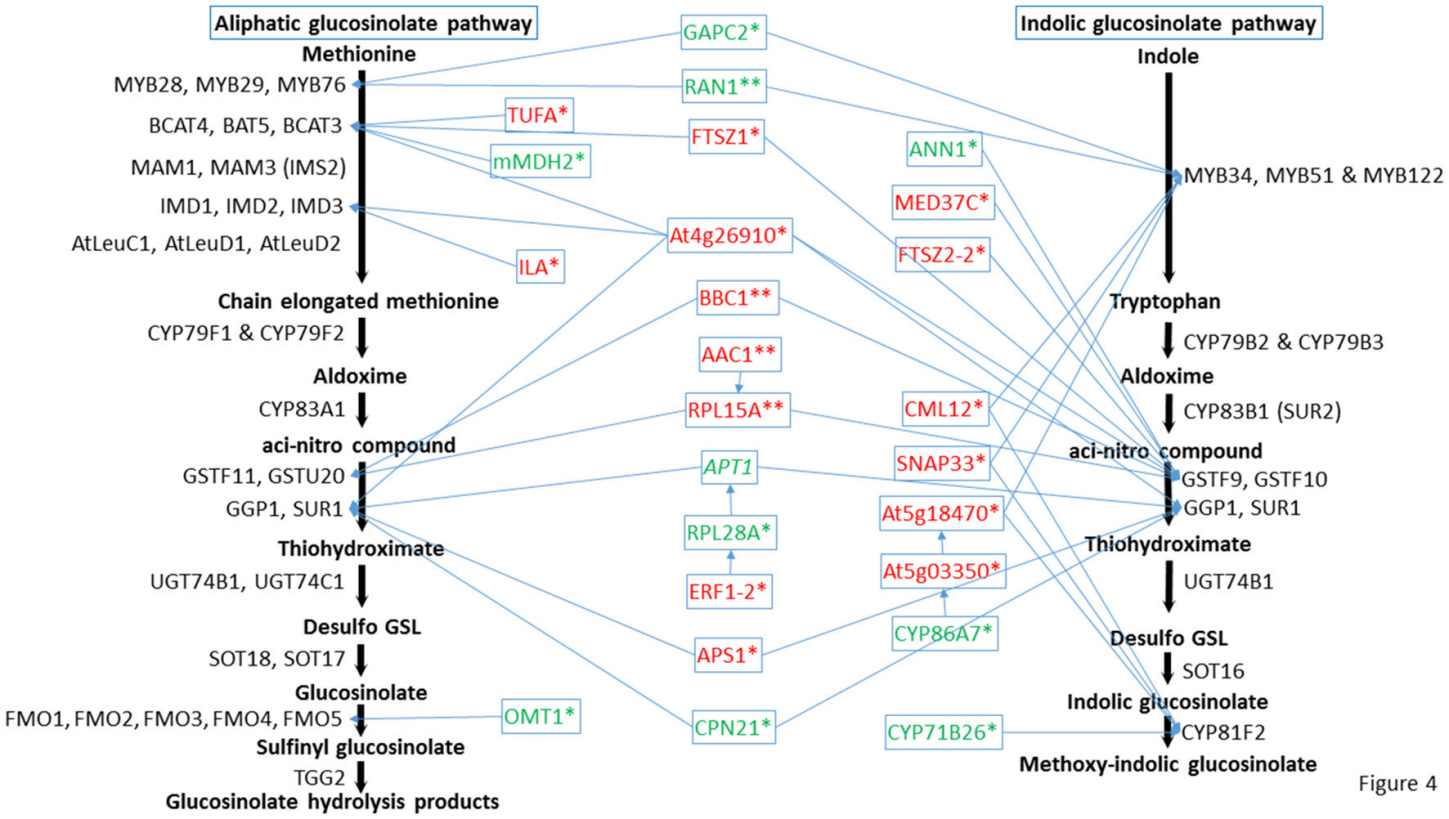
**Predicted positions of the directly connected nodes and connected cytochrome nodes on the glucosinolate metabolic pathway**. Italic indicates proteins changed in both mutants, ^*^ indicates proteins changed in *cyp79B2/B3*, and ^**^ indicates proteins changed in *myb28/29*. Red color means increased proteins, green color means decreased proteins. Full names of the directly connected proteins can be found in the abbreviation and protein name columns in Tables 1–3.

The role of cytochromes P450s 86A7 (CYP86A7) and 71B26 (CYP71B26) in oxidation reduction reaction and oxygen binding (Duan and Schuler, 2005) makes them biologically relevant tertiary nodes in the GMN (Figures 2, 4). Other interesting new tertiary nodes related to Gls metabolism include division protein FtsZ homologs 1 (FTSZ1), 2-1 (FTSZ2-1), 2-2 (FTSZ2-2), curculin-like (mannose-binding) lectin family protein (At5g18470), isoform 3 of dihydrolipoyllysine-residue succinyltransferase component of 2-oxoglutarate dehydrogenase complex 2 (At4g26910), elongation factor Tu (TUFA), ATP sulfurylase 1 (APS1), ABC transporter B family member 26 (ABCB26), flavone 3'-O-methyltransferase 1 (OMT1), Ras-related protein (RABA4A), Ras-related protein (RABB1C), endoglucanase 10 (AT1G75680), dicarboxylate/tricarboxylate transporter (DTC), probable monodehydroascorbate reductase isoform 3 (At3g52880), vesicle transport v-SNARE 11 (VTI11), DnaJ/Sec63 Brl domains-containing protein (ATERDJ2B), and glycosylphosphatidylinositol-anchored lipid protein transfer 6 (At1g55260). Myrosin cells (myrosinase storage sites) endocytosis is controlled by SYP22 from SNARE complex and VPS9A (Shirakawa et al., 2016). Here the decrease of VTI11 from this family is in agreement with the reduced myrosinase, nitrile specifier protein and Gls levels in the soluble proteome (Mostafa et al., 2016) and supports the cross talk between Gls and its hydrolyzing enzymes.

Out of these new nodes, 15 formed direct edges with the GMN: FTSZ1, CML12, FTSZ2-2, At5g18470, At4g26910, TUFA, MED37C, APS1, SNAP33, ILA, GAPC2, OMT1, CYP71B26, ANN1, and mMDH2 in addition to the membrane associated protein (20 kDa chaperonin, CPN21). As we detected a side network correlated to indolic GMN (Mostafa et al., 2016), here we also found a side network strongly correlated to indolic Gls metabolism as it contains nine stress-related proteins out of eleven. These proteins are xyloglucan endotransglucosylase/hydrolase protein 4 (XTH4; Campbell and Braam, 1999), aquaporin PIP2-2 (Javot, 2003; Tournaire-Roux et al., 2003), probable aquaporin PIP1-5 (Weig et al., 1997), aquaporin PIP2-3 (Daniels et al., 1994), probable aquaporin PIP1-4 (Li et al., 2015), plasma membrane intrinsic protein 1B (PIP1B; Alexandersson et al., 2005), aquaporin PIP1-3 (Kammerloher et al., 1994), probable aquaporin PIP2-6 (Alexandersson et al., 2010), and aquaporin PIP2-7 (Weig et al., 1997). Other members in this side network are bifunctional inhibitor/lipid-transfer protein (At2g45180; which has a proteolytic action) and a tetraspanin-18 (TOM2AH2) with unknown functions.

### Specific changes of *myb28/29* membrane proteins

Membrane proteomics of the *myb28/29* mutant showed 28 and 17 proteins to be significantly increased and decreased, respectively (Table 3). STRING analysis of the increased and decreased *myb28/29* specific membrane proteins revealed 21 new nodes in the GMN (Figure 3). Except for the directly connected and stress-related GTP-binding nuclear protein (RAN1; Jiang et al., 2007), other connections including 17 ribosomal proteins [e.g., 60S ribosomal protein L14-2 (RPL14B), 40S ribosomal protein S15-1 (RPS15) and 40S ribosomal protein S15-4 (RPS15D)], and actin-11 (ACT11), ADP/ATP carrier protein 1 (AAC1) and eukaryotic translation initiation factor 3 subunit F (TIF3F1) formed tertiary nodes. These tertiary nodes are connected to the GMN through two bridges (directly connected nodes) which are 60S ribosomal protein L15-1(RPL15A) and 60S ribosomal protein L13-1 (BBC1). The expression changes in ribosomal proteins reflect a correlation between aliphatic Gls perturbation and the translation process in *A. thaliana*.

**Table 3:** **List of membrane proteins the ***myb28/29*** mutant showing significant level changes relative to WT and their biological functions**.

**Accession number**	**Locus tag**	**Protein name**	Abbreviation^*^	**FC^a^**	***p*-value^b^**	**Function**	**TMDs**	**References**
P51422	At3g55750	60S ribosomal protein L35a-4	RPL35AD	1.972	0.025	Translation and RNA binding		Tair
Q9T043	At4g27090	60S ribosomal protein L14-2	RPL14B	1.928	0.040	Translation and RNA binding	D	Tair
Q9LST0	At5g60160	AT5g60160/f15l12_20	AT5G60160	1.686	0.026	Proteolytic activity	D, H, T	Tair
Q9LZ57	At5g02450	60S ribosomal protein L36-3	RPL36C	1.653	0.024	Translation	D	Tair
Q9M0E2	At4g29410	60S ribosomal protein L28-2	RPL28C	1.593	0.009	Translation and RNA binding		Tair
F4I472	At1g04270	40S ribosomal protein S15-1	RPS15	1.501	0.025	Translation	D, H, T	Tair
Q8W463	At4g17560	50S ribosomal protein L19-1	AT4G17560	1.488	0.030	Translation	D, H, T	Tair
Q9FY64	At5g09510	40S ribosomal protein S15-4	RPS15D	1.461	0.036	Translation	D, H, T	Tair
O23515	At4g16720	60S ribosomal protein L15-1	RPL15A	1.417	0.016	Translation		Tair
Q9LZ41	At5g02610	60S ribosomal protein L35-4	RPL35D	1.411	0.010	Translation	D	Tair
F4HRB4	At1g45201	Triacylglycerol lipase-like 1	TLL1	1.374	0.025	Hydrolysis of lipids	D, H, S, T, M	Tair
Q9SUJ1-2	At3g05710	Isoform 2 of Syntaxin-43	SYP43	1.372	0.026	Fungal response and transporter activity	D, H, S, T, M	Zheng et al., 1999; Nielsen and Thordal-Christensen, 2012
Q93VG5	At5g20290	40S ribosomal protein S8-1	RPS8A	1.359	0.028	Translation	D	Tair
B9DGY1	At3g07700	ABC1 kinase	AT3G07700	1.351	0.012	Oxidative stress response	D, H, T, M	Yang et al., 2012
A8MQA1	At3g49010	60S ribosomal protein L13-1	BBC1	1.349	0.041	Translation and RNA binding		Tair
P49693	At4g02230	60S ribosomal protein L19-3	RPL19C	1.331	0.023	Translation and RNA binding		Tair
O22795	At2g33450	50S ribosomal protein L28	RPL28	1.331	0.032	Translation and RNA binding		Tair
Q9C514	At1g48830	40S ribosomal protein S7-1	RPS7A	1.327	0.031	Translation	D	Tair
P49637	At1g70600	60S ribosomal protein L27a-3	RPL27AC	1.274	0.006	Translation and RNA binding		Tair
F4IHJ8	At2g21580	40S ribosomal protein S25-2	AT2G21580	1.273	0.032	Translation	D	Tair
P53496	At3g12110	Actin-11	ACT11	1.267	0.014	Cytoskeleton component, Binding of ATP	D, T	McDowell et al., 1996; Jia et al., 2013
Q9FH02	At5g42270	ATP-dependent zinc metalloprotease FTSH 5	FTSH5	1.240	0.012	Leaf coloration and photo-inhibition	D, H, T	Sakamoto et al., 2002
P31167	At3g08580	ADP, ATP carrier protein 1	AAC1	1.238	0.015	Transport activities	D, H, T, M	Tair
P51418	At2g34480	60S ribosomal protein L18a-2	RPL18AB	1.229	0.028	Translation		Tair
Q9LVI9	At3g17810	Putative dehydrogenase	PYD1A	1.224	0.016	Oxidation reduction reactions, pyrimidine and uracil metabolism	D, T	Zrenner et al., 2009
Q8RWA5	At1g25380	Nicotinamide adenine dinucleotide transporter 2	NDT2	1.223	0.043	Transport activities	D, H, T	Bedhomme et al., 2005
Q8W486	At1g04910	O-fucosyltransferase family protein	AT1G04910	1.213	0.042	Glycosyl groups transfer	D, H, S, T, M	Voxeur et al., 2012
P51427	At3g11940	40S ribosomal protein S5-2	RPS5B	1.206	0.002	Translation and RNA binding	D, T	Tair
O04202	At2g39990	Eukaryotic translation initiation factor 3 subunit F	TIF3F1	0.799	0.029	Translation and development of embryo	D, T	Xia et al., 2010
Q9FQ25	At3g55000	Protein TONNEAU 1a	TON1A	0.782	0.046	Cell division and cytoskeleton organization	D, T	Azimzadeh et al., 2008
Q84LG4	At3g09800	Coatomer subunit zeta-2	AT3G09800	0.773	0.049	Transport of protein	D, H, T	Tair
P41916	At5g20010	GTP-binding nuclear protein Ran-1	RAN1	0.770	0.002	Salt stress response and GTP binding	D, T	Jiang et al., 2007
Q9ZVA2	At1g78830	At1g78830/F9K20_12	F9K20.12	0.767	0.017	Binding of carbohydrate	D, H, S, T, M	Tair
Q9FIX1	At5g39730	AIG2-like protein	AT5G39730	0.740	0.021	Salt stress response	D	Tair
Q9LS26	At5g46570	At5g46570	BSK2	0.740	0.036	Kinase activity	D, T	Tair
Q93ZH0-2	At1g21880	Isoform 2 of LysM domain-containing GPI-anchored protein 1	LYM1	0.738	0.005	Immunity and defense activity	D, H, S, T, M	Willmann et al., 2011
Q94EG6	At5g02240	Uncharacterized protein	AT5G02240	0.734	0.040	Abscisic acid response	D, T	Ghelis et al., 2008
Q0WSY2	At1g19835	Filament-like plant protein 4	FPP4	0.725	0.036		D	Tair
Q9C500	At1g47200	WPP domain-containing protein 2	WPP2	0.716	0.049	Growth of lateral roots and mitotic division	D	Patel, 2004
Q9FIJ2	At5g47890	NADH dehydrogenase 1 alpha subcomplex subunit 2	AT5G47890	0.690	0.050	Oxidation reduction reactions	Mitochondrial membrane	Michalecka et al., 2003, Murray et al., 2003
Q9FPJ4	At5g47200	Ras-related protein RABD2b	RABD2B	0.687	0.013	Binding of GTP	D, T	Tair
Q94F08	At5g62630	HIPL2 protein	HIPL2	0.678	0.004	Binding of carbohydrate and oxidation reduction reaction	D, H, S, T	Tair
Q9SZ51	At4g31840	Early nodulin-like protein 15	ENODL15	0.666	0.017	Stimuli response and electron carrier	D, H, S, T, M	Tair
P48421	At4g13770	Cytochrome P450 83A1	CYP83A1	0.664	0.033	Glucosinolate biosynthesis, insect response	D, H, S, T, M	Mostafa et al., 2016
Q9SK39	At2g24940	Probable steroid-binding protein 3	MP3	0.376	0.031	Binding of steroid and heme	Membrane associated	Tair, Yang et al., 2005

* *Abbreviations for unique proteins in Figure 3*.

a *Fold change at cut-off point >1.2 or < 0.8*.

b *p < 0.05*.

*TMDs, transmembrane domains; D, Das; H, HMMTOP; S, SOSUI; T, TMPred; M, TMHMM*.

### Gene ontology analysis of the significantly changed membrane proteins

AgriGO enrichment analysis of the changed proteins was conducted at the biological processes (BP), cellular components (CC), and molecular functions (MF) levels. By annotating 147 changed membrane proteins in the *cyp79B2/B3* using SEA, we got 302 enriched GO terms for BP (Supplementary Figure 1), 63 for CC (Supplementary Figure 2), and 47 for MF (Supplementary Figure 3). SEA of 54 changed membrane proteins in the *myb28/29* showed 45 enriched GO terms for BP (Supplementary Figure 4), 56 for CC (Supplementary Figure 5) and 2 for MF (Supplementary Figure 6). SEACOMPARE of the mutant revealed 271 BP, 21 CC, and 46 MF GO terms to be enriched specifically in *cyp79B2/B3*, while 14 BP, 14 CC, and one MF were the specifically enriched GO terms in *myb28/29* (Supplementary Table 4). From this BP analysis, it was obvious that responses to stimuli including abiotic, chemical and stress were highly enriched in *cyp79B2/B3* in addition to transport, photosynthesis and metabolic processes. In *myb28/29*, the most enriched BP terms were those related to translation process. This observation supported our results concerning the stimuli and translation-related proteins in the *cyp79B2/B3* and *myb28/29*, respectively (Supplementary Table 4). On the level of CC, the high enrichment of membrane GO terms supported the effectiveness of our membrane preparation procedure (Supplemental Figures 2, 5).

### Comparison of protein expression data with transcription data

To determine whether protein level changes correlated with gene transcription changes, we examined the transcript levels of 32 and 22 genes from *cyp79B2/B3* and *myb28/28*, respectively (Supplementary Table 5). The two mutants exhibited different patterns of correlation. In comparison of *cyp79B2/B3* to WT, the genes investigated showed a positive correlation between transcript and protein levels in both direction and degree of expression (*r* = 0.6579, *p* = 4.269e^−05^; Supplementary Figure 7). However, in comparison of *myb28/29* to WT, the genes did not show correlation between the transcript and protein levels (*r* = 0.0887, *p* = 0.6945; Supplementary Figure 7), only three out of the 22 genes showed similar regulation at both transcript and protein levels. For example, At4g13770 encoding cytochrome P450 83A1, exhibited down-regulation in *myb28/29* compared to WT (Supplementary Table 5). The difference in the degree of correlation in these two mutants implies that different regulatory mechanisms are involved in the transcriptional and posttranscriptional processes in different genotypes (Marmagne et al., 2010; Koh et al., 2012).

## Discussion

As a result of Gls metabolism perturbation, many changes in the levels of soluble (Mostafa et al., 2016) and membrane proteins took place. It was interesting to discover new cytochromes to be involved in the GMN. In addition, several groups of stress and defense-related proteins as well as binding and transport activity proteins were related to the indolic and aliphatic GMNs, in addition to a group of ribosomal proteins in the *myb28/29* mutant.

### Three new cytochromes in the glucosinolate molecular network

**Figure 5:**
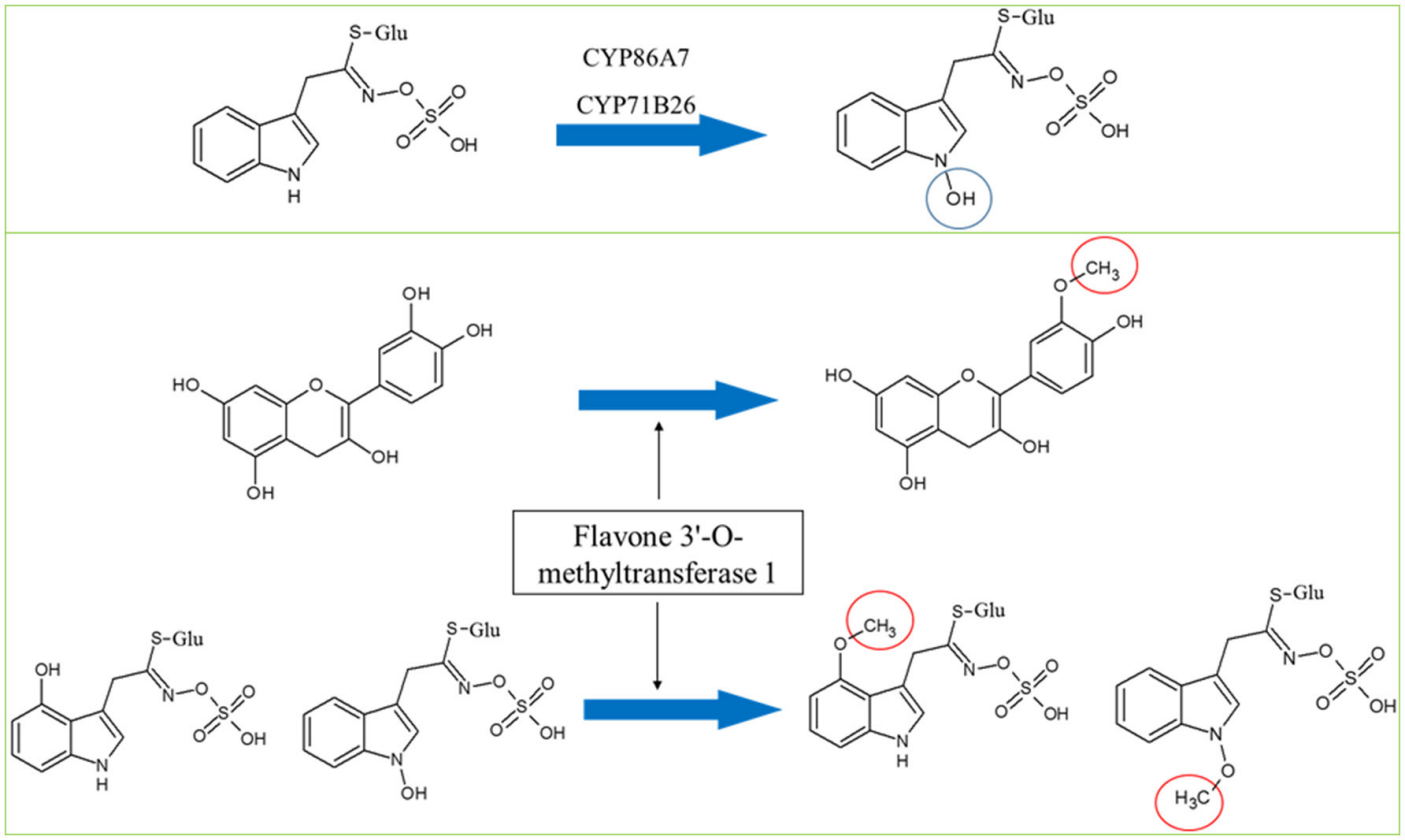
**Hypothesized roles of CYP86A7 and CYP71B26 in the hydroxylation of indolic-1-glucosinolate (top panel) and the potential dual functions of flavone 3′-O-methyltransferase in flavonoid and Gls metabolism**. Circles indicate chemical modifications to the substrates.

Cytochromes play a key role in Gls biosynthesis. In aliphatic Gls biosynthesis, CYP79F1 and CYP79F2 catalyze the conversion of chain-elongated methionines to aldoximes, which are metabolized by another cytochrome (CYP83A1) to *aci*-nitro compounds, precursors of desulphoglucosinolates (Grubb and Abel, 2006). As to indolic Gls biosynthesis, CYP79B2 and CYP79B3 convert tryptophan to aldoximes, that are metabolized by CYP83B1 to form the *aci*-nitro compounds (Grubb and Abel, 2006). In addition, there is another CYP81F2 catalyzing the conversion of indolic-3-glucosinolate to 4-hydroxy-indolic-3-glucosinolate (Sønderby et al., 2010). Furthermore, CYP71A12 and CYP71A13 can metabolize indolic aldoximes to indole acetonitrile and subsequently indole acetic acid derivatives (Nafisi et al., 2007). In our previous study, we reported cytochrome B5 isoform C and cytochrome c oxidase subunit 5b-2 to be new nodes in the aliphatic and indolic GMNs, respectively (Mostafa et al., 2016). Here we discovered cytochrome P450 86A7 (CYP86A7) in redox reaction and metabolism of fatty acids (Duan and Schuler, 2005), and cytochrome P450 71B26 (CYP71B26) as new nodes in the indolic GMN. Based on STRING analysis, CYP71B26 is connected to CYP81F2 through a direct edge, while CYP86A7 is connected indirectly to CYP81F2 through lectin family proteins (At5g03350 and At5g18470; Figures 2, 4). Given that their connection to a specific and key enzyme in indolic Gls biosynthetic pathway (CYP81F2) and their expression levels were decreased in the *cyp79B2/B3* mutant (Table 2), it is reasonable to hypothesize that CYP86A7 and CYP71B26 play specific roles in 4-hydroxy indolic-3-glucosinolate production (Figure 4). Especially their precursor (indolic-3-glucosinolate) and the product were decreased in *cyp79B2/B3* mutant as revealed in our previous study (Mostafa et al., 2016). Also by similarity, we can predict a role for the enzymes in hydroxy indolic-1-glucosinolate production (Figure 5) as its synthesizing enzymes are not known (Sønderby et al., 2010). The third new cytochrome discovered in this study is a probable cytochrome c At1Gg22840 (CYC2), which plays a role in electron transport process (Welchen et al., 2012). CYC2 is in the shared decreased protein category, forming new connections with aliphatic GMN through ADP/ATP carrier protein 1 (AAC1) and 60S ribosomal protein L15-1 (RPL15A), which is connected to GSTF9, GSTF10 and GSTF11, and with indolic GMN through eukaryotic peptide chain release factor subunit 1–2 (ERF1-2), 60S ribosomal protein L28-1 (RPL28A) and adenine phosphoribosyltransferase 1 (APT1). APT1 is connected to GGP1 and SUR1. Although the CYC2 function awaits for further studies, it might play a role in the conversion of *aci*-nitro compounds to thiohydroximates.

### Stress related membrane protein changes as a secondary result of glucosinolate metabolism perturbation

Plant Gls metabolism is responsive to stress conditions, e.g., temperature and light stress (Martínez-Ballesta et al., 2013), water stress (Khan et al., 2010), salt stress (Guo et al., 2013), and microbial stress (Clay et al., 2009). In our previous study, glucan endo-1,3-beta-glucosidase, glutathione S-transferase F2 and glutathione S-transferase F7 in addition to others as stress-related proteins were found to connect to the Gls pathway (Mostafa et al., 2016). Here we found the levels of 51 stress-related proteins changed significantly in the *cyp79B2/B3* mutant and six with changes in the *myb28/29* mutant. In the *cyp79B2/B3* membrane proteome, a group of general stimuli response-related proteins exhibited significant changes compared to WT (Table 2). Among them, the following are examples to directly connect with Gls enzymes: calmodulin-like protein 12 (CML12; Cazzonelli et al., 2014; connected to the indolic GMN via MYB122 and CYP81F2), mediator of RNA polymerase II transcription subunit 37c (MED37C; Lee et al., 2009; connected via GSTF9 to GMN, with possible role in thiohydroximate formation), and glyceraldehyde-3-phosphate dehydrogenase (GAPC2; Guo et al., 2012; formed edges with GMN through MYB28, MYB29, MYB76, and MYB34, suggesting roles in methionine chain-elongation and tryptophan synthesis; Figures 2, 4). It is known that GAPC2 participates in the oxidation of glyceraldehydes-3-phophate to glycerate from which pyruvate is formed. The pyruvate can be converted to acetylCoA for methionine chain-elongation in aliphatic Gls biosynthesis or for synthesis of tryptophan in indolic Gls pathway (Mann, 1987). Both glucosinolate classes were decreased in the *cyp79B2/B3* mutant in our previous study (Mostafa et al., 2016) together with *GAPC2* in this study. Therefore, the connection between GAPC2 and MYBs in the STRING maps reflects functional relationship and does not necessarily indicate direct physical interaction. Another stress related group showing expression level changes was the salt stress and water deficiency group represented by chloride channel protein CLC-c (Jossier et al., 2010), aquaporin PIP2-2 (Javot, 2003; Tournaire-Roux et al., 2003), annexin D1 (ANN1; Gorecka et al., 2005; Jia et al., 2015; formed edge with GSTF9), early-responsive to dehydration stress protein (Rai et al., 2016), probable aquaporin PIP1-5 (Weig et al., 1997), aquaporin PIP2-3 (Daniels et al., 1994), probable aquaporin PIP1-4 (Li et al., 2015), plasma membrane intrinsic protein 1B (Alexandersson et al., 2005), aquaporin PIP1-3 (Kammerloher et al., 1994), probable aquaporin PIP2-6 (Alexandersson et al., 2010), and aquaporin PIP2-7 (Weig et al., 1997; Figures 2, 4). The decreased expression of this group of aquaporins (Table 2) confirms crosstalk between indolic Gls production and water deficiency enzymes (Khan et al., 2010). The mechanism underlying such crosstalk is intriguing. The reduction in aquaporins potentiates our observation of retarded growth of Gls mutants (Mostafa et al., 2016). The decreased Gls production resulted in stress status, which led to decreased water uptake and decreased expression of aquaporins, and thus growth retardation.

The immunity and defense process was also affected by Gls perturbation, and it is represented by changes in the directly connected nodes: SNAP25 homologous protein (SNAP33; Eschen-Lippold et al., 2012; connected by MYB51 in tryptophan synthesis and CYP81F2 to GMN), protein ILITYHIA (ILA; Monaghan and Li, 2010; playing a role in methionine chain elongation by forming edges with IMD1, IMD2, and IMD3) and a 20 kDa chaperonin (CPN21; Takáč et al., 2014; connected to GMN by the edge GGP1; Figures 2, 4). Another protein exhibiting expression changes and connected to GMN is malate dehydrogenase 2 (mMDH2), which participates in bacterial defense (Jones et al., 2006; Figures 2, 4). In *myb28/29*, a GTP-binding nuclear protein Ran-1 (Jiang et al., 2007) was found to connect MYB28, MYB29, MYB76, MYB34, MYB51, and MYB122, suggesting its role in methionine chain-elongation and tryptophan synthesis (Figures 3, 4 and Table 3).

### Effects of glucosinolate metabolism perturbation on other processes and nodes

Gls biosynthetic pathway is organelle specific and involves transport starting from methionine chain-elongation, sulfate transport, and ending with Gls storage in the seeds (Sønderby et al., 2010; Gigolashvili and Kopriva, 2014; Jørgensen et al., 2015). Here we report a decrease in ABC transporter B family member 19 (Lin and Wang, 2005) in both mutants (Table 1). In addition to their role in sulfate transport, ABC transporters are involved in transporting Gls hydrolysis products (Kang et al., 2011). This result indicates the decrease in glucosinolate levels in the mutants feedback regulate the ABC transporter level. In *cyp79B2/B3*, a curculin-like (mannose-binding) lectin family protein (At5g18470) involved in carbohydrate binding forms connections with MYB51 and CYP81F2 (Figures 2, 4). How this lectin family protein function is not known. Another biological process affected by the Gls perturbation is photosynthesis as revealed by the increase of photosystem I reaction center subunit IV B in both mutants (Table 1), and increases in *cyp79B2/B3* photosystem II stability/assembly factor HCF136 (Meurer et al., 1998), protein curvature thylakoid 1B, NAD(P)H-quinone oxidoreductase subunit H, light-harvesting complex I chlorophyll a/b binding protein 1 and light-harvesting chlorophyll protein complex II subunit B1 (Table 2). The increased activity in the photosynthetic process could be a strategy to compensate for the internal stress in the mutants as indicated by changes of many stress-related proteins (Tables 2, 3; Mostafa et al., 2016). It was obvious that aliphatic Gls metabolism perturbation activated the ribosomal protein expression as reflected by the increased levels of 18 ribosomal proteins in the *myb28/29* (Table 3). The biological implication of this change is not known although we can correlate it to the regulation of aliphatic Gls biosynthetic pathway by MYB28 and MYB29 (Li et al., 2013).

In both mutants, adenine phosphoribosyltransferase 1 (APT1) acting on adenine phosphorylation (Allen et al., 2002) showed connections with GGP1 and SUR1, so it might have a role in thiohydroximate formation (Figures 2–4). Its decrease in levels may be a feedback of the decreased Gls production in the mutants. In *cyp79B2/B3*, FtsZ homolog 1 (FTSZ1) involved in chloroplast division and protein binding (Osteryoung et al., 1998) was found to connect with BCAT3 and GSTF9, suggesting it may affect methionine chain-elongation and thiohydroximate synthesis. Interestingly, another FtsZ homolog 2-2 (FTSZ2-2; McAndrew et al., 2008) was also connected with GSTF9 (Figures 2, 4). Isoform 3 of dihydrolipoyllysine-residue succinyltransferase component of 2-oxoglutarate dehydrogenase complex 2 (At4g26910) is a member of tricarboxylic acid cycle and can affect methionine biosynthesis and its coupling to acetylCoA in the chain elongation process. Interestingly, it was found to form multiple connections with GMN via BAT5, BCAT3, IMD1, IMD2, IMD3, GSTF9, and SUR1 (Figures 2, 4). In addition, ATP sulfurylase 1 (APS1), a hydrogen sulfide biosynthesis enzyme, formed edges with GGP1 and SUR1, suggesting its potential role in thiohydroximate synthesis (Figures 2, 4). The increased levels of the aforementioned proteins may reflect a feedback mechanism to compensate for reduced Gls levels in the *cyp79B2/B3*. Flavone 3'-O-methyltransferase 1 (OMT1) in flavonoid metabolism (Muzac et al., 2000) was connected with FMO1, so it could participate in sulfinyl Gls formation (Figures 2, 4). This finding provides another line of evidence for the pathway interaction between phenylpropanoids and glucosinolates. Previously, methionine derived aldoximes were shown to directly or indirectly inhibit caffeic acid O-methyltransferase (COMT) and caffeoyl-CoA O-methyltransferase CCoAOMT), leading to low levels of phenylpropanoid metabolites (Hemm et al., 2003). Here the decreased levels of OMT1 in *cyp79B2/B3* may contribute to the decreased production of sulfinyl Gls in the mutant. The data support our metabolomics finding concerning the decreased shikimate level (Mostafa et al., 2016). Another possibility of the OMT1 activity is methylation of hydroxy-indolyl Gls to form methylated indolic Gls (unknown before, Sønderby et al., 2010) in a way similar to methylation of quercetin into isorhamnetin (Figure 5). In *myb28/29*, 60S ribosomal proteins L13-1 (BBC1) and L15-1 (RPL15A) might be a component in thiohydroximate synthesis through the connections with GSTF9, GSTF10 and/or GSTF11. Both proteins were increased, presumably to compensate for the deficiency of aliphatic Gls in the mutant (Mostafa et al., 2016).

### The proteome and transcriptome correlation

In the *cyp79B2/B3*, the defense and stress-related genes calreticulin 3 (At1g08450; Sun et al., 2014), calmodulin (At2g41100; Cazzonelli et al., 2014), lectin (At5g03350; Armijo et al., 2013), and SNAP25 (At5g61210; Eschen-Lippold et al., 2012) showed significant upregulation in the transcriptome and increases in the proteome. Malate dehydrogenase 2 expression was decreased at both the transcript and protein levels, and it is known to be involved in bacterial defense (Jones et al., 2006). These data have provided additional evidence for the relationship between indolic glucosinolates and stress responses. The overall positive correlation between protein and gene expression levels in the *cyp79B2/B3* indicates transcriptional regulation of indole glucosinolates. In *myb28/29*, although there was no overall correlation between transcript and protein levels, isoform 2 of LysM (At1g21880; Willmann et al., 2011) and AIG2 (avirulence induced gene, At5g39730) exhibited similar downregulation patterns as their corresponding proteins. Both genes are involved in cellular stress responses (Jiang et al., 2007; Willmann et al., 2011). Post-transcriptional and post-translational regulations may contribute to the non-correlation between the expression of some of the genes and their encoded proteins in *myb28/29*.

## Conclusions

Glucosinolate biosynthetic process is controlled by several cytochrome proteins known to be localized to the membrane, but little is known about how Gls metabolism would affect the membrane proteome. In this study, we aim to address this important question utilizing the TMT labeling based quantitative proteomics of two genetic mutants, i.e., *cyp79B2/B3* as the indolic Gls mutant and *myb28/29* as the aliphatic Gls mutant. We identified 4,673 proteins, out of which 2,171 were membrane proteins. From these membrane proteins and after transmembrane domain analysis, 192 exhibited different levels relative to WT, with cytochrome P450 86A7, cytochrome P450 71B26 and probable cytochrome c representing new cytochromes potentially involved in GMN. Based on our analyses, the first two might play a role in hydroxyl-indolic Gls production. In addition, a flavone 3′-O-methyltransferase 1 is hypothesized to participate in the methylation process of the hydroxyl-indolic Gls to form methoxy-indolic Gls. GO functional enrichment revealed important processes related to stress response, transport activities and photosynthesis in the *cyp79B2/B3* and those related to protein translation in the *myb28/29*. A transcription profiling of both mutants showed a strong correlation between transcript and protein levels in *cyp79B2/B3*, and no significant correlation in *myb28/29*. Overall, the new nodes and edges discovered in the GMNs are useful resources for future hypothesis-testing experiments and ultimately toward engineering and breeding of Gls profiles with positive impacts on human health and plant defense.

## Author contributions

IM performed the experiments, data analysis and paper drafting; MY performed qRT-PCR experiment and data analysis; NZ participated in protein extraction and peptides labeling; SG conducted the statistical analysis; CD contributed in LC/MS analysis of peptides; MA and ME provided supervision and advice, and SC designed the experiments, supervised the work and finalized the manuscript.

### Conflict of interest statement

The authors declare that the research was conducted in the absence of any commercial or financial relationships that could be construed as a potential conflict of interest. The reviewer XH and handling Editor declared their shared affiliation, and the handling Editor states that the process nevertheless met the standards of a fair and objective review.

## Supplementary material

Supplementary Figure 1 **Biological process GO enrichment of membrane proteins differentially expressed in *cyp79B2/B3* compared to WT**.

Supplementary Figure 2 **Cellular component GO enrichment of membrane proteins differentially expressed in *cyp79B2/B3* compared to WT**.

Supplementary Figure 3 **Molecular function GO enrichment of membrane proteins differentially expressed in *cyp79B2/B3* compared to WT**.

Supplementary Figure 4 **Biological process GO enrichment of membrane proteins differentially expressed in *myb28/29* compared to WT**.

Supplementary Figure 5 **Cellular component GO enrichment of membrane proteins differentially expressed in *myb28/29* compared to WT**.

Supplementary Figure 6 **Molecular function GO enrichment of membrane proteins differentially expressed in *myb28/29* compared to WT**.

Supplementary Figure 7 **Correlation between transcript and protein levels inferred from 32 to 22 genes for *cyp79B2/B3* and *myb28/29*, respectively**. Pearson correlation *r* = 0.6579 (*p* = 4.269e^−05^) for *cyp79B2/B3* and *r* = 0.0887 (*p* = 0.6945) for *myb28/29*.

Supplementary Table 1 **Primer information used in qRT-PCR**.

Supplementary Table 2 **Proteomics data from two independent experiments (the data were generated using Proteome Discoverer 1.4 by searching the raw data against the Arabidopsis tair 10 database)**.

Supplementary Table 3 **Transmembrane domains predection analyses**.

Supplementary Table 4 **GO enrichment of proteins differentially expressed in *cyp79B2/B3* and *myb28/29* mutants relative to WT using AgriGO SEACOMPARE**.

Supplementary Table 5 **Gene expression at transcript and protein levels in *cyp79B2/B3* and *myb28/29* relative to WT**.

## Abbreviations

Gls: glucosinolate
GMN: glucosinolate molecular network
GO: Gene Ontology
TMT: tandem mass tags
WT: *Arabidopsis thaliana* wild type.
