# Supplementary figures and images for: Membrane Proteomics of Arabidopsis Glucosinolate Mutants cyp79B2/B3 and myb28/29
Source: Front Plant Sci. 2017 Apr 11;8:534. doi: 10.3389/fpls.2017.00534 (PMC5387099; doi:10.3389/fpls.2017.00534)

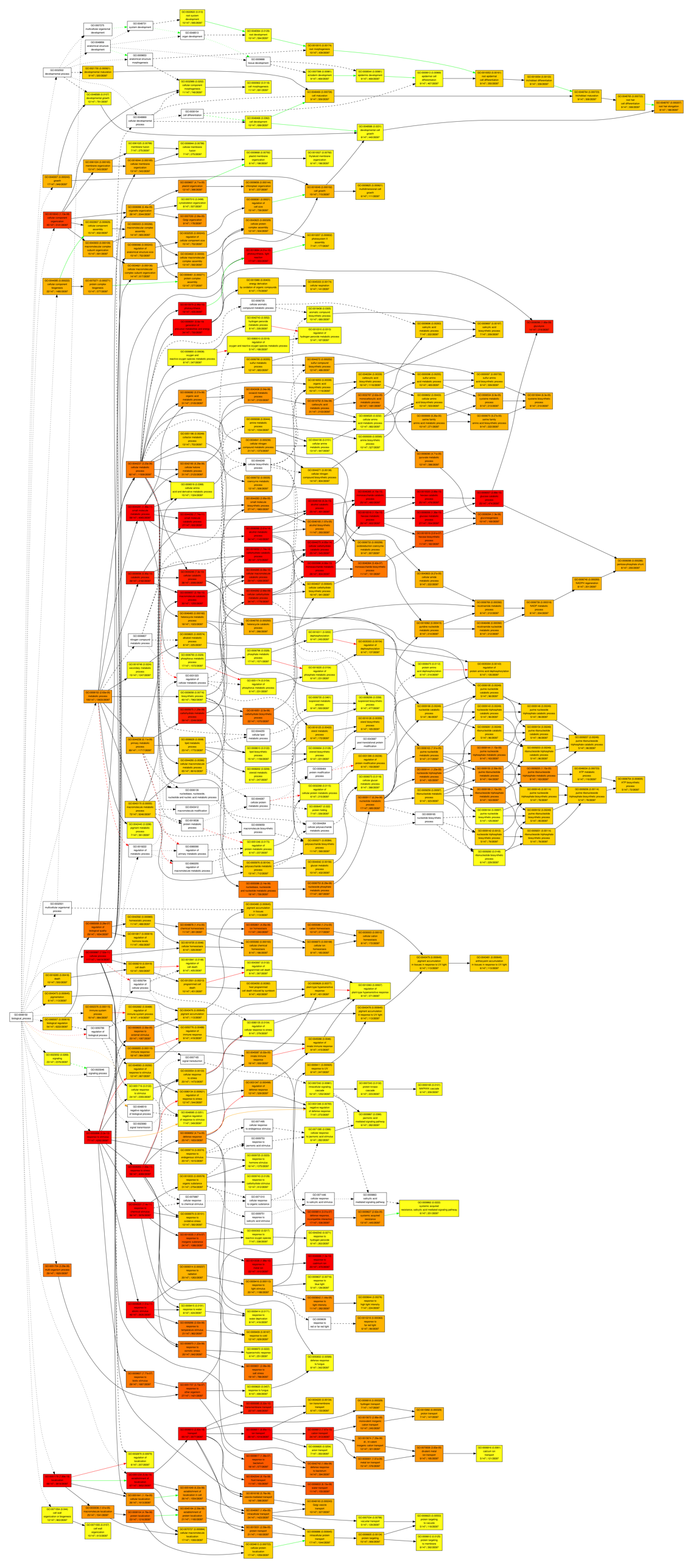

Supplement: Supplementary Figure 1 — Biological process GO enrichment of membrane proteins differentially expressed in cyp79B2/B3 compared to WT. [file Image1.PDF]

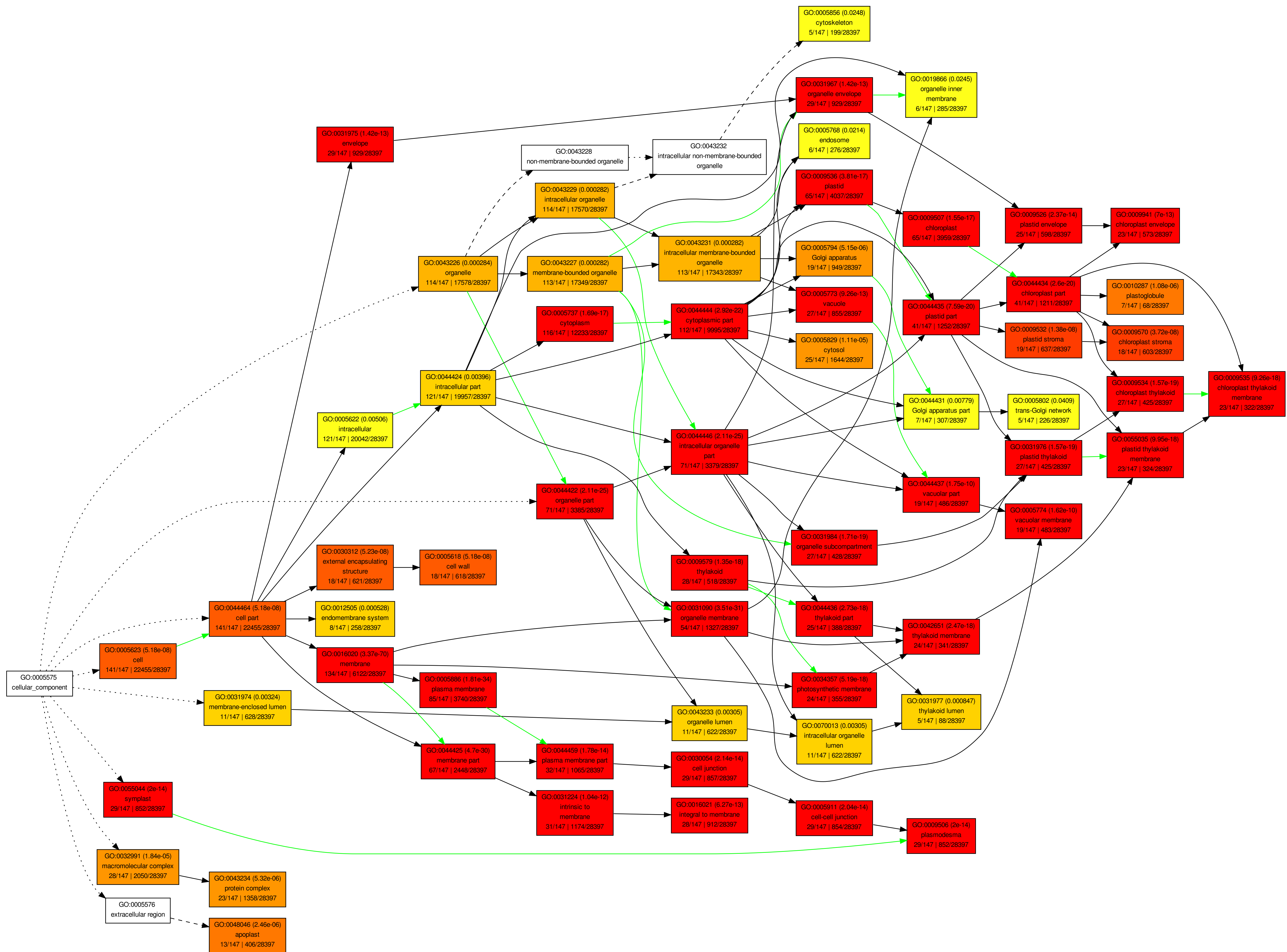

Supplement: Supplementary Figure 2 — Cellular component GO enrichment of membrane proteins differentially expressed in cyp79B2/B3 compared to WT. [file Image2.PDF]

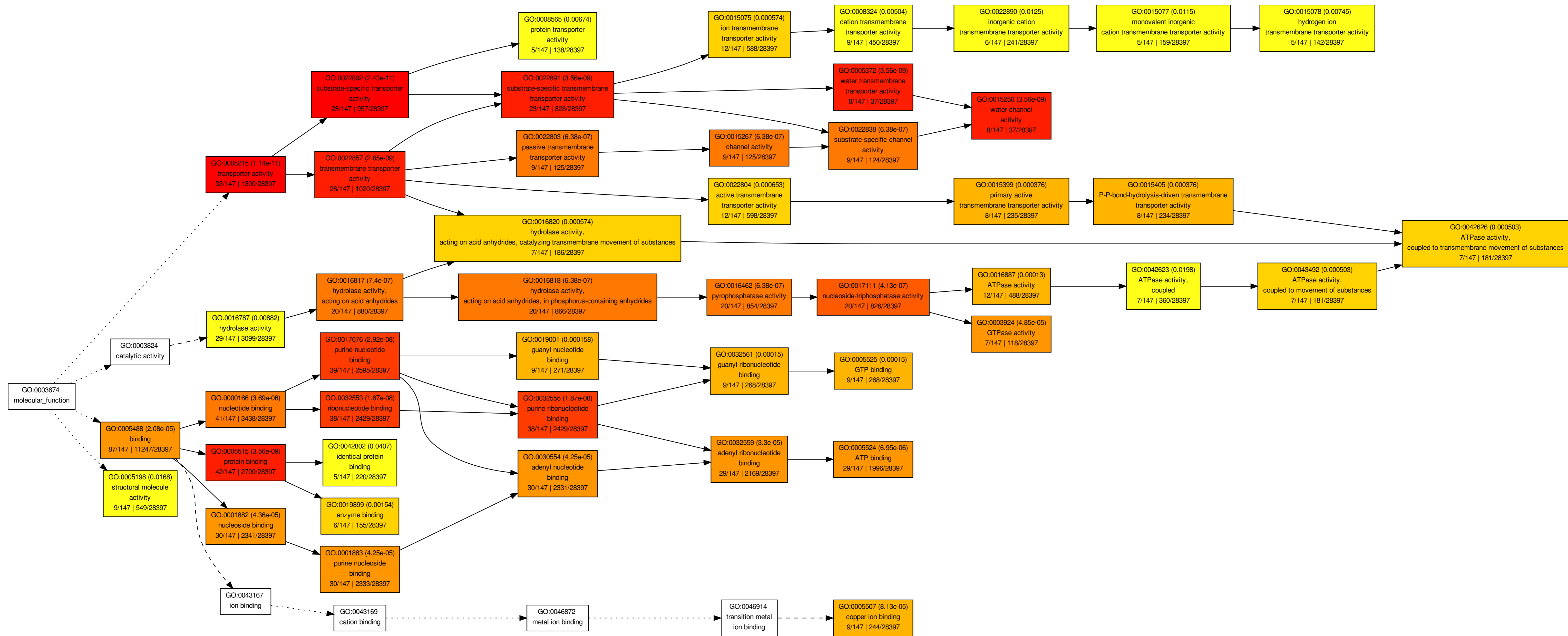

Supplement: Supplementary Figure 3 — Molecular function GO enrichment of membrane proteins differentially expressed in cyp79B2/B3 compared to WT. [file Image3.PDF]

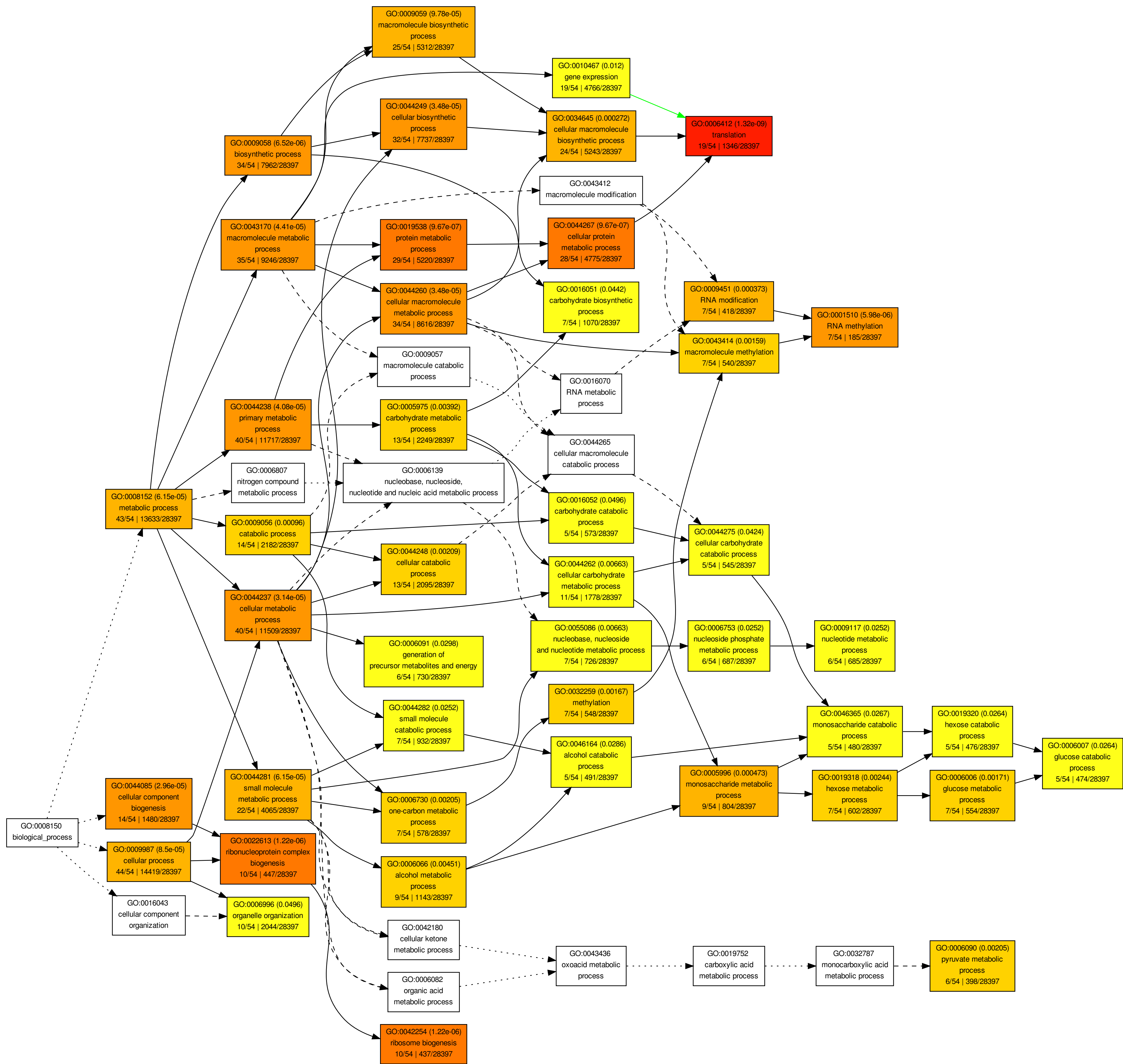

Supplement: Supplementary Figure 4 — Biological process GO enrichment of membrane proteins differentially expressed in myb28/29 compared to WT. [file Image4.PDF]

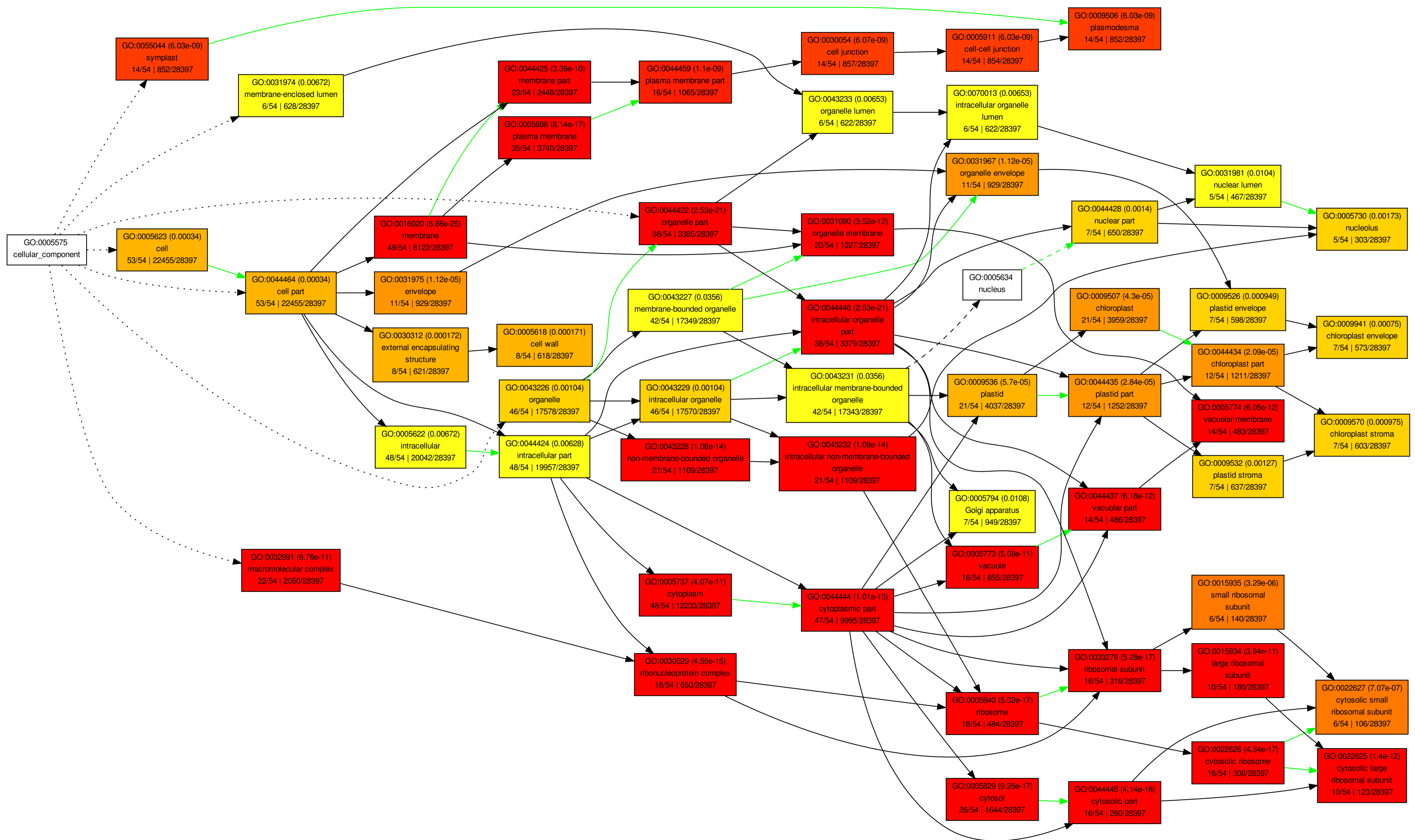

Supplement: Supplementary Figure 5 — Cellular component GO enrichment of membrane proteins differentially expressed in myb28/29 compared to WT. [file Image5.PDF]

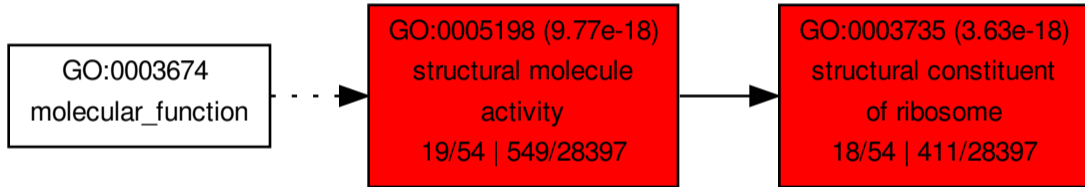

Supplement: Supplementary Figure 6 — Molecular function GO enrichment of membrane proteins differentially expressed in myb28/29 compared to WT. [file Image6.PDF]
