# Supplementary material for: Membrane Proteomics of Arabidopsis Glucosinolate Mutants cyp79B2/B3 and myb28/29
Source: Front Plant Sci. 2017 Apr 11;8:534. doi: 10.3389/fpls.2017.00534 (PMC5387099; doi:10.3389/fpls.2017.00534)
Supplement: Supplementary Figure 7 — Correlation between transcript and protein levels inferred from 32 to 22 genes for cyp79B2/B3 and myb28/29, respectively. Pearson correlation r = 0.6579 (p = 4.269e−05) for cyp79B2/B3 and r = 0.0887 (p = 0.6945) for myb28/29. [file Image7.PDF]

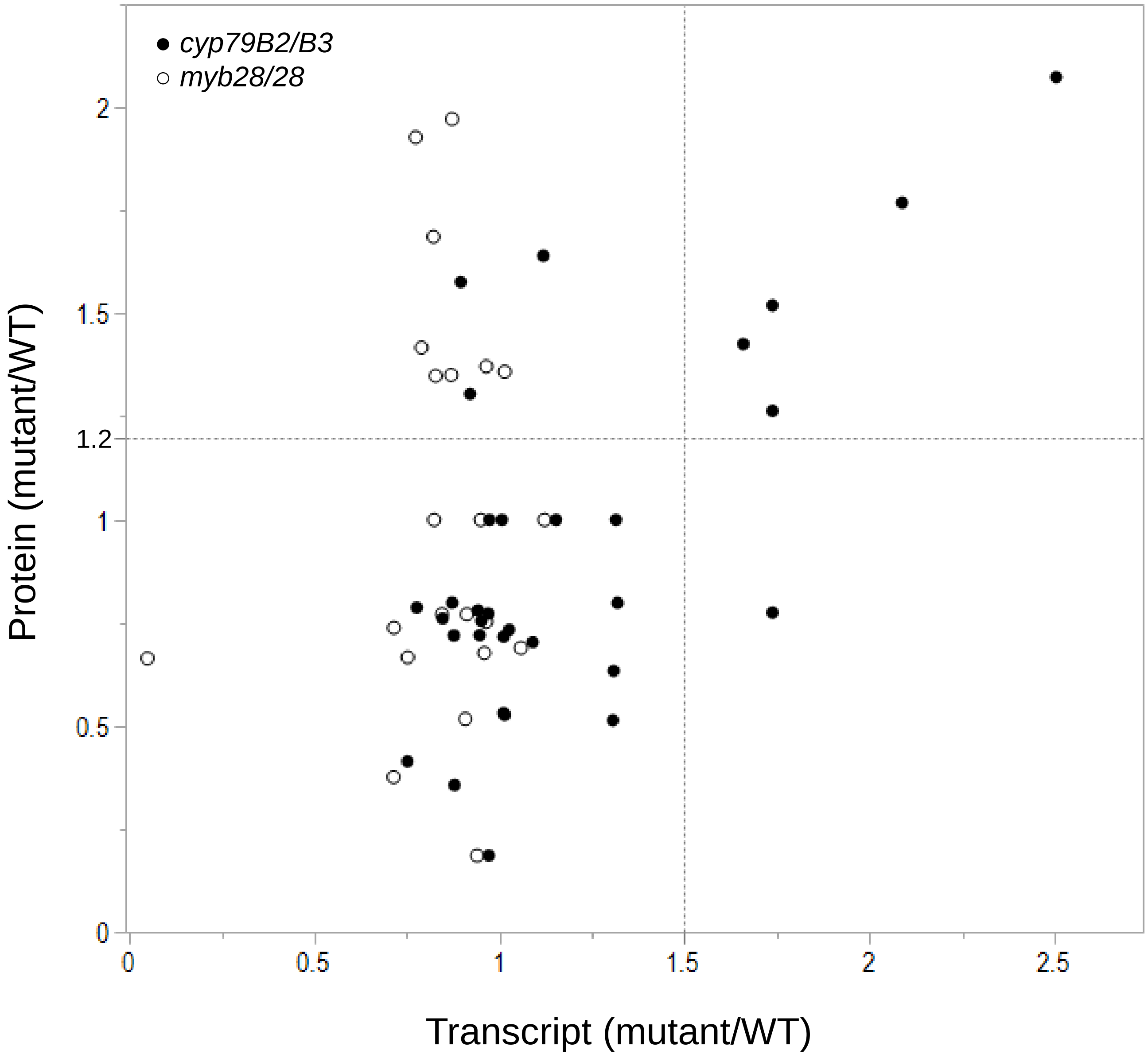

Supplemental figure 7. Correlation between transcript and protein levels inferred from 32 and 22 genes for *cyp79B2/B3* and *myb28/29*, respectively. Pearson correlation r = 0.6579 (p-value =4.269e^{-05}) for *cyp79B2/B3* and r = 0.0887 (p-value = 0.6945) for *myb28/29*.
